# Single-cell multi-omics analysis revealed the expansion of age-associated B cells in the pancreas of type 1 autoimmune pancreatitis patients

**DOI:** 10.1186/s13073-025-01567-w

**Published:** 2025-11-04

**Authors:** Jiaxin Wang, Chenxiao Liu, Xianda Zhang, Tianyi Che, Yizhou Zhao, Qidi Yang, Xianzheng Qin, Yifei Chen, Xiang Ao, Xiaonan Shen, Xiangyi He, Tingting Gong, Ling Zhang, Minmin Zhang, Dong Wang, Yanhua Du, Li Wen, Youqiong Ye, Yao Zhang, Chunhua Zhou, Duowu Zou

**Affiliations:** 1 Department of Gastroenterology, Ruijin Hospital, Shanghai Jiao Tong University School of Medicine, Shanghai, 200025 China; 2 Shanghai Institute of Immunology, Department of Immunology and Microbiology, Shanghai Jiao Tong University School of Medicine, Shanghai, 200025 China; 3 Institute of Clinical Medicine, Peking Union Medical College Hospital, Chinese Academy of Medical Science & Peking Union Medical College, Beijing, 100730 China

**Keywords:** Type 1 autoimmune pancreatitis, IgG4-related disease, Single-cell RNA sequencing, Age-associated B cells, CXCL9, T follicular helper cells

## Abstract

**Background:**

Type 1 autoimmune pancreatitis (AIP) is pancreatic manifestation of IgG4-related disease (IgG4-RD), characterized by pancreatic lymphoplasmacytic infiltration. Despite this well-known pathological feature, the immune microenvironment and the complex cellular interactions within the pancreas in AIP remain poorly understood. This study aimed to characterize the local immune features of the pancreas in AIP patients.

**Methods:**

We employed single-cell RNA sequencing (scRNA-seq), immune receptor repertoire sequencing (scTCR/BCR-seq), and spatial transcriptome sequencing on biopsy samples from lesion tissues of AIP patients. Flow cytometry, multicolour immunofluorescence, and functional assays were performed to validate the findings from bioinformatics analysis.

**Results:**

Our results revealed an increased presence of IgD^−^ age-associated B cells (ABCs) in the pancreas of AIP patients. These ABCs were predicted to differentiate into plasma cells that secrete IgG. Additionally, *CXCL9*^+^ macrophages were found to recruit IgD^−^ ABCs via the CXCL9-CXCR3 axis. Elevated levels of T follicular helper cells (Tfhs) were also observed, which interacted with IgD^−^ ABCs through IL-21 secretion. Both ABCs and Tfhs were localized at the periphery of pancreatic tertiary lymphoid structures (TLSs). Importantly, these immune abnormalities were specific to AIP and were not present in the pancreases of patients with chronic pancreatitis.

**Conclusions:**

These findings highlight significant alterations in the pancreatic immune microenvironment in AIP and propose a potential pathogenic model involving ABCs, Tfhs, and macrophages. This model provides valuable insights that could inform the development of targeted therapeutic strategies for AIP.

**Supplementary Information:**

The online version contains supplementary material available at 10.1186/s13073-025-01567-w.

## Background

Autoimmune pancreatitis (AIP) is a distinctive type of chronic pancreatitis (CP) driven by autoimmune responses, categorized into two subtypes: type 1 and type 2 [1]. Type 1 AIP constitutes the majority of global cases and is also the pancreatic manifestation of IgG4-related disease (IgG4-RD) characterized by elevated levels of IgG4 in both the serum and affected organs [2, 3]. For clarity, type 1 AIP will be referred to as AIP throughout this article. Glucocorticoids are currently the first-line treatment for AIP, though they are prone to high relapse rates and can lead to significant side effects, including infections, diabetes, osteoporosis, and cataracts [4, 5]. Moreover, AIP presents additional therapeutic challenges such as steatorrhea, diabetes, local complications, and psychosocial issues [6]. Therefore, a deep understanding of AIP’s pathogenesis is essential for discovering new treatment strategies.

Research has highlighted the critical roles of various immune cells such as activated B-cells, T follicular helper cells (Tfhs), CD4^+^ cytotoxic T lymphocytes, and M2 macrophages in the immune dysregulation seen in IgG4-RD. Key cytokines, including interleukin-4, interleukin-1, interleukin-33, and interferon-gamma (IFN-γ), are also crucial in this process [2, 7–10]. Elevated levels of these cells and cytokines have been noted in the peripheral blood and pancreatic tissues of AIP patients [4, 11, 12]. However, our comprehension of the immune microenvironment and complex cellular interactions within the pancreas in AIP remains incomplete.

Advancements in high-throughput single-cell RNA sequencing (scRNA-seq) and spatial transcriptomics (ST) sequencing have greatly enhanced our ability to analyse cellular diversity in disease progression [13]. For instance, scRNA-seq of submandibular gland tissues from IgG4-RD patients has provided insights into T-B-cell interactions in affected tissues [14]. Similarly, a study by Bomi Lee et al. compared immune transcriptomic features in hereditary and idiopathic chronic pancreatitis, revealing an upregulated CCR6-CCL20 axis in hereditary cases [15]. Nonetheless, a comprehensive examination of the immune microenvironment in the AIP pancreas and its differences from IgG4-RD and CP remain elusive. This study aims to use scRNA-seq and ST of pancreatic tissues from AIP patients to identify specific immune cell subpopulations and clarify their roles in AIP pathogenesis.

## Methods

### Human specimens

Human pancreatic tissues used for scRNA-seq were collected from two groups via endoscopic ultrasound-guided fine-needle aspiration/biopsy (EUS-FNA/B) from Ruijin Hospital: specifically, the inflamed pancreatic regions of 10 patients with AIP, and the adjacent normal non-inflamed pancreatic tissues of 8 patients with pancreatic cystic lesions (PCLs). All enrolled AIP patients had diffuse pancreatic lesions and required histopathological evidence via EUS-FNA/B to establish a definitive diagnosis, in accordance with the International Consensus Diagnostic Criteria (ICDC) for type 1 AIP [1]. During the sample collection procedure, an experienced endoscopist performed endoscopic ultrasound (EUS) after administering intravenous anaesthesia. A 22G Franseen needle (Acquire, Boston Scientific, Natick, MA) was used for sampling, with the puncture site selected based on the presence or absence of jaundice. Once the rapid on-site evaluation confirmed sample quality, an additional puncture was conducted for research purposes. For patients with PCLs, a 22G Expect needle (Boston Scientific, Natick, MA) was employed. The initial puncture was used to obtain adjacent normal pancreatic tissue, while a subsequent puncture was performed to collect cystic fluid for clinical diagnosis. An additional cohort comprising 5 AIP patients and 5 PCLs patients were enrolled and we collected their pancreatic tissues for flow cytometry following the same procedure described above. From these participants, we also collected their peripheral blood samples for chemotaxis assay and enzyme-linked immunosorbent assay (ELISA).

Pancreatic samples for immunofluorescence or ST were collected from 5 AIP patients, 10 CP patients, and 5 PCLs patients who underwent total pancreatectomy from Ruijin Hospital. The initial suspicion was pancreatic ductal adenocarcinoma (PDAC) before surgery; however, AIP or CP was confirmed by pathology following the procedure. Their peripheral blood samples were also collected for ELISA.

All biopsies and surgeries occurred before a definitive diagnosis was established, ensuring that all participants were treatment-naive at the time of sampling. All procedures for sample collection adhered to standard clinical practices. The demographics and characteristics of control and AIP patients included in the scRNA-seq analysis (cohort 1) are detailed in Table 1 and Additional file 1: Table S1. For the validation experiments (cohorts 2 and 3), demographic information for control, CP, and AIP patients is provided in Tables 2 and 3 and Additional file 1: Table S2-S3.

**Table 1:** Statistical comparisons of demographic data and patient characteristics between non-inflamed control (NI) group and AIP patients for scRNA-seq analysis (cohort1)

	NI (*n* = 8)	AIP (*n* = 10)	*P* value
Age (y/o, mean ± SD)	65.8 ± 10.7	66.7 ± 8.6	0.84
Sex (male/female)	3/5	10/0	0.0065
IgG4 (g/L, mean ± SD)	0.8 ± 0.3	7.9 ± 6.2	0.0057
TBIL (μmol/L, mean ± SD)	13.5 ± 2.7	57.5 ± 66.8	0.08
Body mass index (kg/m^2^, mean ± SD)	21.5 ± 1.7	21.0 ± 2.5	0.66

*y/o* years old, *TBIL* total bilirubin

**Table 2:** Statistical comparisons of demographic data and patient characteristics among NI group and AIP patients for flow cytometry experiments (cohort2)

	NI (*n* = 5)	AIP (*n* = 5)	*P* value
Age (y/o, mean ± SD)	68.8 ± 4.4	68.4 ± 4.5	0.89
Sex (male/female)	2/3	5/0	0.17
IgG4 (g/L, mean ± SD)	0.7 ± 0.3	13.2 ± 3.5	< 0.0001
TBIL (μmol/L, mean ± SD)	31.9 ± 18.4	70.8 ± 92.5	0.38
Body mass index (kg/m^2^, mean ± SD)	21.8 ± 2.9	20.7 ± 1.1	0.48

*y/o* years old, *TBIL* total bilirubin

**Table 3:** Statistical comparisons of demographic data and patient characteristics among NI group, CP and AIP patients for immunofluorescence or ST experiments (cohort 3)

	NI (*n* = 5)	CP (*n* = 10)	AIP (*n* = 5)	*P* value
Age (y/o, mean ± SD)	59.2 ± 11.8	55.5 ± 13.4	68.4 ± 9.2	0.18
Sex (male/female)	4/1	8/2	5/0	0.78
IgG4 (g/L, mean ± SD)	0.9 ± 0.3	0.4 ± 0.4	12.0 ± 8.2	0.0002
TBIL (μmol/L, mean ± SD)	26.3 ± 6.9	13.1 ± 5.0	40.0 ± 31.0	0.020
Body mass index (kg/m^2^, mean ± SD)	24.5 ± 2.6	22.2 ± 4.2	23.7 ± 4.1	0.51

*y/o* years old, *TBIL* total bilirubin

### Tissue processing and single-cell isolation

Fresh pancreatic tissues were preserved in 2x protease inhibitor (Solarbio, P6730) mixed with complete RPMI 1640 medium (containing 10% foetal bovine serum (FBS), 100 U/mL penicillin, and 100 mg/mL streptomycin) on ice for transport. Tissue processing began within 30 min of tissue acquisition after EUS-FNA/B. Upon arrival, the tissues were washed with ice-cold phosphate-buffered saline (PBS) and cut into 2-mm pieces. Each sample was then enzymatically digested using a solution of 1 mg/mL Trypsin inhibitor (Sigma, T6522), 0.82 mg/mL Dispase (Sigma, D4693), 1 mg/mL collagenase VIII, and 0.15 mg/mL DNase I in 4 mL complete RPMI 1640 medium. Digestion was stopped by adding 4 mL of complete RPMI 1640 medium. Red blood cells were lysed with 2 mL ACK lysing buffer (Thermofisher, A1049201) for 5 min on ice. After two washes with 1 × PBS (Corning), the cell pellets were resuspended in single-cell buffer. The cell suspensions were filtered through a 70-μm cell strainer (BD) and 1% protease inhibitor was added. The freshly prepared single-cell suspensions were then used for scRNA-seq and flow cytometry.

### Flow cytometry

Fresh cells from AIP pancreatic tissues and non-inflamed tissues were washed and incubated with Live/Dead dye (BV510, Biolegend, 1:1000) in PBS for 10 min at 4 °C. After incubation, cells were washed in PBS with 2% FBS and 2 mM ethylenediaminetetraacetic acid (EDTA), and this solution was defined as fluorescence-activated cell sorting (FACS) buffer. To minimize non-specific protein binding, myeloid cells were treated with human Fc Block (1:100) at 4 ℃ for 20 min. Surface markers were then stained with a panel of antibodies diluted in FACS buffer at the specified concentrations. The staining was conducted at 4 °C for 30 min using the following antibodies: Anti-CD45 (BD Horizon, 563792, 1:200), anti-CD4 (Biolegend, 317434, 1:200), anti-CD19 (Biolegend, 363024, 1:200), anti-CD3(BD Horizon, 563725, 1:200); anti-CD11c (Biolegend, 301644, 1:50), anti-IgD (Biolegend, 348240, 1:100), anti-PD-1 (Biolegend, 329910, 1:200), anti-CD38 (Biolegend, 303524, 1:200), anti-CD27 (Biolegend, 302815, 1:200). After staining, the cells were washed twice. Cells were then fixed (Fixation Buffer, BD), permeabilized (Intracellular Staining Permeabilization Wash Buffer (10X), BD) and subjected to intracellular labelling using anti-CD68 (Biolegend, 333806, 1:200). Flow cytometry was performed on a BD Symphony (BD Biosciences) with data acquisition managed by BD FACSDiva software v8.0.2 and analysis performed using FlowJo v.10.81 (Tree Star Inc.). Statistical comparisons were carried out using a paired two-tailed *t*-test with GraphPad Prism 6.

### Multiplex immunofluorescent (mIF) staining

Human pancreatic tissue samples were fixed in 4% paraformaldehyde for 24 h, followed by dehydration and paraffin embedding. They were then sectioned into 5-μm slices for use. Multiplex immunofluorescence was performed using PanoPANEL Kits (Panovue, 10234100050) according to the manufacturer’s instructions. The procedure began with deparaffinization of the slides using xylene and a series of ethanol washes (100%, 95%, and 70%). This was followed by microwave-based antigen retrieval and a 30-min antibody blocking step. Primary antibodies were incubated at room temperature for 1 h, followed by incubation with HRP-labelled secondary antibodies for 30 min at room temperature. The slides were then treated with Tyramide signal amplification (TSA) fluorescent dye working solution for 30 min. The following antibodies and corresponding fluorescent dyes were used for mIF staining: panel1: CD19 (Abcam, ab134114, 1:500, PPD480); CD11c (Abcam, ab52632, 1:500, PPD520); CD4 (Abcam, ab133616, 1:500, PPD570); PD1 (Abcam, ab52587, 1:100, PPD650); CD23 (Abcam, ab16702, 1:100, PPD780); panel2: CD68(Abcam, ab955,1:1000, PPD520); CXCL9 (Abcam, ab290643, 1:100, PPD650). After staining, nuclei were counterstained with DAPI for 20 min, and the slides were then sealed with Nail Polish. Scanning of the slides was performed using the SLIDEVIEW VS200 (Olympus).

### HALO spatial analysis

Images from mIF were analysed with HALO software (Indica Labs, Corrales, NM, USA). Quantification of cells and staining was performed according to HALO HighPlex FL analysis instructions. The Tissue Classifier function was employed to distinguish between tertiary lymphoid structures (TLSs) and non-TLS areas using the Random Forest algorithm, as illustrated in Fig. 5d. And the infiltration of age-associated B cells (ABCs) and Tfhs to peripheral boundary of TLS was evaluated by using infiltration analysis related to Fig. 5e. Proximity analysis was conducted to evaluate (1) the percentage of ABCs (CD19^+^CD11c^+^) within 100 μm (divided into 10 equal ranges) of Tfhs (PD1^+^CD4^+^) related to Fig. 5f and (2) the percentage of ABCs located within or outside a 50-µm radius of Tfhs, as depicted in Fig. 5g.

### B cell subset isolation

B cells were isolated from the peripheral blood of AIP patients or healthy people using MojoSort™ Human B Cell (CD43^−^) Isolation Kit (Biolegend, 480061) according to the manufacturer’s instructions. The purity of isolated cells was verified to be more than 95%. The collected cells were used for functional chemotaxis and co-culture experiments.

### Macrophages culture

THP-1 cells (Quicell, Quicell-T239) were plated at 1 × 10^5^ per well of 24-well plates and cultured in RPMI-1640 supplemented with 10% FBS, 1% penicillin/streptomycin, and 50 mM β-mercaptoethanol at 37 °C in a humidified atmosphere of 5% CO_2_. THP-1 derived M1-type macrophages were obtained by culturing THP-1 cells with 100 ng/ml phorbol ester (PMA) (Beyotime, S1819) for 24 h and subsequently stimulated with 100 ng/ml lipopolysaccharides (LPS) (MCE, HY-D1056) for 24 h.

### Transfection

THP-1 derived M1-type macrophages were cultured to reach the confluency of 70% before the transfections. Negative control or CXCL9-targeting small interfering RNA (siRNA) were prepared and mixed with lipofectamine RNAiMAX (Invitrogen, 13778150) according to the manufacturer’s instruction. After the addition of siRNA-lipofectamine RNAiMAX, cells were incubated for a further 24 h before chemotaxis experiments.

### Reverse transcription-quantitative polymerase chain reaction (RT-qPCR)

Total RNA was extracted from THP-1 derived M1-type macrophages transfected with either si-negative control or si-CXCL9 using Trizol (Invitrogen). cDNA was then synthesized from SuperScript IIIcDNA Synthesis Kit (Invitrogen). mRNA expressions were detected with SYBR Green on a 384 well real-time PCR system (Applied Biosystems, QuantStudio 6 Flex). Primer sequences were listed as follows: CXCL9, Antisense 5′—CCAGTAGTGAGAAAGGGTCGC – 3′, and Sense5′- AGGGCTTGGGGCAAATTGTT −3′; GAPDH, Antisense5′- ACAACTTTGGTATCGTGGAAGG −3′, and Sense 5′- GCCATCACGCCACAGTTTC −3′; siCXCL9, Antisense 5’—UUUCUCACUACUGGGGUUCTT—3′, and Sense 5′- GAACCCCAGUAGUGAGAAATT—3′. We utilized 2^−ΔΔCt^ method to calculate the normalized expression of each gene relative to gene GAPDH.

### Chemotaxis assay

Isolated B cells from the peripheral blood of AIP patients were resuspended at 1 × 10^6^ cells/ml in preheated RPMI1640 supplemented with 0.5% BSA. For anti-CXCR3 group, the B cells were incubated for 30 min at 37 °C with anti-CXCR3 blocking antibody (Biolegend, 353702, 1:100). Then, in the lower chamber of the Transwell apparatus (Corning, Transwell-24 well with 5-µm pore polycarbonate membrane, #3421), 600 µl of recombinant human CXCL9 (Peprotech, 300-26-5UG) was added to achieve a final concentration of 100 ng/ml. In the upper chamber, 200 µl of cell suspension was added. Wells with added vehicle served as negative controls. For macrophages-B cell chemotaxis assay, Transwell apparatus (Corning, Transwell-24 well with 5-µm pore polycarbonate membrane, #3421) were inserted into the 24-well plates with the si-negative control or si-CXCL9 transfected macrophages cultured in. Two hundred microlitres of B cell suspension was added into the upper chamber. After incubating at 37 °C for 3 h, migrating cells were collected from the lower chamber and evaluated by flow cytometry according to the protocol described above. All assays were performed in triplicate.

### T cell subsets isolation and co-culture experiment

T cells were isolated from the peripheral blood of AIP patients using FACS Arial III (BD Bioscience) and sorted as PD1^+^CD4^+^Tfhs and PD1^−^CD4^+^T helper cells (Ths). The collected cells were used for functional co-culture experiments. Sorted Th and Tfhs were plated at 1 × 10^4^ cells per well of 96-well round-bottom plate and cultured in RPMI-1640 supplemented with 10% FBS and 1% penicillin/streptomycin. They were then stimulated with CD3/CD28 beads (Easylso, AH2001) or 50 ng/ml IL-21R-Fc (UA BIOSCIENCE, UA011187) for 48 h and further co-cultured with isolated healthy blood B cells at a ratio of 1:5 in the final volume of 200 µL for 6 days. Cells were collected and evaluated by flow cytometry according to the protocol described above.

### ELISA detection

The peripheral blood of AIP, CP, and control group patients was centrifuged at 1800 rpm (757 g) and room temperature for 5 min. The plasma was obtained for the ELISA detection. Fifty microlitres of plasma from each sample was transferred to a 96-well plate for the detection of CXCL9 using Human CXCL9 ELISA Kit (ml060599, Mlbio) following the manufacturer’s protocol. Finally, the absorbance at 450 nm was measured using a Multiskan Go (Thermofisher).

### Single-cell RNA-seq, TCR/BCR-seq, and data processing

Single-cell suspensions were prepared according to the manufacturer’s protocol for the 10x Chromium Next GEM Single Cell 5’ Kit v2 (10x Genomics, PN-1000263) to generate libraries for single-cell transcriptome and TCR/BCR V(D)J analysis. Sequencing was then conducted on an Illumina NovaSeq 6000 platform at the Shanghai Institute of Immunology. Sequence processing was performed using the standard Cell Ranger pipeline (10x Genomics), with data mapped to the GRCh38 reference genome using Cell Ranger Software (v5.0.1). The resulting preliminary count matrices were analysed with Seurat (v4.3.0) [16]. Genes expressed in fewer than three cells were removed, and cells with over 10% mitochondrial genes, 25% red cell genes, fewer than 200 or more than 7000 gene counts, or UMI counts outside the range of 500 to 50,000 were filtered out. Potential doublets were identified and removed using Scrublet (v0.2.3) and DoubletDetection (v3.0) [17, 18]. The data was then normalized and transformed to a log scale using the “NormalizeData” function.

### Spatial transcriptomics sequencing and data processing

Formalin-fixed paraffin-embedded (FFPE) 5-μm pancreatic lesion tissue sections from a representative AIP patient were used. Haematoxylin and eosin (H&E) staining was performed on the sections firstly. The sections were then de-crosslinked and hybridized with transcriptomic probes to detect the presence of target RNA. After probe hybridization, the Visium CytAssist system was used to transfer these transcriptomic probes from the glass slides to the Capture Area on the Visium slide. Following this, library preparation and sequencing were carried out using the 10x Visium CytAssist Spatial Gene Expression for FFPE, Human Transcriptome, 6.5 mm kit (10x Genomics, PN-1000520) according to the manufacturer’s protocol. Sequencing was performed on an Illumina NovaSeq 6000 platform at Xu Ran Biological (Shanghai, China). Raw sequencing reads from the ST data were quality-checked and mapped to the GRCh38 reference genome for human samples using Space Ranger (v2.1.0).

### Dimension reduction and unsupervised clustering

The top 4000 most variable genes were identified using the “FindVariableFeatures” function. Gene expression levels were then scaled by regressing out unwanted sources of variation, including total counts, mitochondrial gene counts, and ribosomal gene counts. These variable genes were used for principal component analysis (PCA), and the top 20 principal components were selected for subsequent analyses. Graph-based clustering was performed using the “FindNeighbors” and “FindClusters” functions to identify cell subtypes. Dimensionality reduction was achieved using Uniform Manifold Approximation and Projection (UMAP). For unsupervised clustering, cells were initially categorized into major types, including T cells, NK cells, B cells, plasma cells, myeloid cells (monocytes, macrophages, dendritic cells, and neutrophils), acinar cells, ductal cells, proliferating cells, fibroblasts, endothelial cells, and endocrine cells, based on canonical cell markers. Subsequently, specific subtypes within B cells, CD4^+^ T cells, CD8^+^ T cells, and macrophages were further resolved at higher resolutions. Any unexpected gastric and duodenal epithelial cells introduced during the EUS-FNA/B sampling procedure were removed from the analysis.

### Analysis of differentially expressed genes

Differentially expressed genes (DEGs) between clusters were identified using the “FindAllMarkers” function with a minimum percentage threshold of 0.1 and a log fold-change threshold of 0.25. DEGs were visualized using a heatmap after log transformation and scaling of gene expression data. The Wilcoxon rank-sum test Find Clusters was applied to calculate *p*-values for the comparisons, and *p*-values were adjusted for multiple testing using the Bonferroni correction. A comprehensive list of canonical and signature marker genes for each cell cluster is provided in Additional file 1: Table S4-8.

### Functional annotation of genes and Gene Set Enrichment Analysis (GSEA) analysis

Gene Ontology biological process (GO BP) analysis was performed using the clusterProfiler package (v4.0.5) [19] to conduct functional enrichment analyses of DEGs. The analysis utilized a *p*-value to assess the probability of observing pathway members, based on an accumulative hypergeometric distribution. TLS scores were calculated using the ing the f observing pathway members, based on an accumulative hypergeometric distribution. nature marker genes for each cell cluste [20] and are listed in Additional file 1: Table S9. Additionally, gene sets from the C5: ontology gene set collection in The Molecular Signatures Database (MSigDB, http://software.broadinstitute.org/MSigDB/msigdb/) [21] were downloaded. GSEA was performed using the GSEA function in the clusterProfiler package (v4.0.5) [19] to identify significantly differentially enriched annotated gene sets between macrophages from the AIP group and those from the non-inflamed group. Gene signatures or pathways with a false discovery rate (FDR) less than 0.05 were considered significantly enriched.

### Trajectory analyses

Developmental trajectory analyses of ABCs and plasma G cells were realized with Monocle2 (v2.22.0) [22]. The top 2000 highly variable genes in these cells were selected as input for the analysis. Dimensionality reduction was performed using the “DDRTree” method. Differentially expressed genes along the pseudotime trajectory were identified with the “differentialGeneTest” function, applying a *q*-value threshold of < 0.01. The results were visualized using the “plot_pseudotime_heatmap” function.

### Cell–cell interaction analysis

Intercellular communication of ligand-receptor pairs was analysed using CellChat (v1.1.3) [23]. The CellChatDB.human database was utilized for signalling pathways. The communication probability between interacting cell clusters was calculated using the “computeCommunProb” function. The “netVisual_x” functions were employed to visualize the strength of cell–cell communication networks and significant ligand-receptor pairs. Additionally, NicheNet (v2.0.0) [24] was used to infer crosstalk between CD4^+^ T cells and IgD^−^ ABCs. For ligand-receptor interactions, genes expressed in more than 10% of the cells within each cluster were considered. CD4^+^ T cells were designated as sender cells, while IgD^−^ ABCs were considered as affected cells, with other B cell subsets used as reference cells. The top 10 ligands from the sender cells and the top 250 targets of differentially expressed genes in the affected cells were selected for paired ligand-receptor activity analysis. The regulatory activity of ligands was visualized using the “ligand_activity_target_heatmap” function in the NicheNet output.

### Single-cell TCR/BCR-seq analysis

TCR and BCR clonotype assignment was performed using the Cell Ranger (v5.0.1, GRCh38 reference genome) VDJ pipeline (10x Genomics) to obtain diversity metrics, including clonotype frequency and barcode information. Subsequent analysis was conducted using the scRepertoire package (v2.0.0) [25]. For BCR analysis, each cell was assigned the pair of heavy and light chains with the highest UMI counts. The strict definition of a clone is based on the normalized Levenshtein edit distance of CDR3 nucleotide sequences and V-gene usage via the function “combineBCR”. For TCR analysis, each cell was assigned the pair of alpha and beta chains with the highest UMI counts. Unique CDR3 amino acid sequences were defined as clones, and cells with identical clones were considered to have originated from the same ancestor. TCR/BCR clones were correlated with scRNA-seq data and projected onto UMAP plots based on barcode information. To analyse the origin of plasma cells, the assigned the pair of alpha and beta chains with the highest UMI counts. Unique CDR3 amino acid sequences were defined as clones, and cells with identical clones function. The “ClonalDiversity” function assessed TCR/BCR clonotype diversity using metrics such as Shannon entropy, inverse Simpson index, normalized entropy, Gini-Simpson index, Chao1, and ACE. Additionally, the “clonalOccupy” and “clonalOverlap” functions were used to evaluate the clonal size and overlap of each T cell or B cell subpopulation, respectively.

### Spatial deconvolution and annotation in ST

To determine the cell composition of spots on spatial transcriptomics slides, we utilized the Cell2location Python package (v0.1.3) [26]. The analysis began with preprocessing the raw data, including standard quality control and filtering to exclude low-quality spots and genes with minimal expression. The data was then normalized using the total-count normalization method provided by Cell2location. We trained the Cell2location model using our integrated scRNA-seq atlas alongside the ST data. This model predicted the expression of each gene for each cell type at each spot in the tissue, enabling us to identify and visualize the spatial distribution of *CXCL9*^+^ macrophages, IgD^−^ ABCs and Tfhs within the tissue. Additionally, we used the ding standard quality control and filtering to exclude low-quality spots and genscore.

### Tissue distribution of immune cell subsets among AIP, CP, and NI groups

ScRNA-seq data of CP were acquired from the Gene Expression Omnibus (GEO) database at https://www.ncbi.nlm.nih.gov/geo/ in GSE165045 [27]. To eliminate the batch effect, we performed harmony algorithm in R package Harmony v0.1.0 [28] to remove batch effect before integrating analysis. To compare the immune microenvironment among AIP, CP, and control groups, we utilized the “FindTransferAnchors” and “TransferData” functions in Seurat. This approach involved evaluating the cell composition of the pancreas from CP patients by using scRNA-seq data from CP as the query set and our dataset as the reference. We calculated the ratio of observed to expected cell numbers (Ro/e) for each cell subset across different tissues (AIP, CP, and non-inflamed) to quantify the preference of each subset. The expected cell numbers for each cell subset and tissue combination were derived from the *χ*^2^ test. A cell subset was considered enriched in a specific tissue if RO/E was greater than 1.

### Statistical analysis

Statistical analyses were conducted using R or GraphPad Prism 6, with a significance level set at *P* < 0.05. The methods used included two-tailed *t*-tests, Wilcoxon rank-sum tests, and one-way analysis of variance (ANOVA), as specified in the figure legends. For multiple comparisons, Bonferroni correction was applied.

## Results

### Single-cell transcriptome analysis revealed the landscape of the pancreatic microenvironment in human AIP patients

**Fig. 1:**
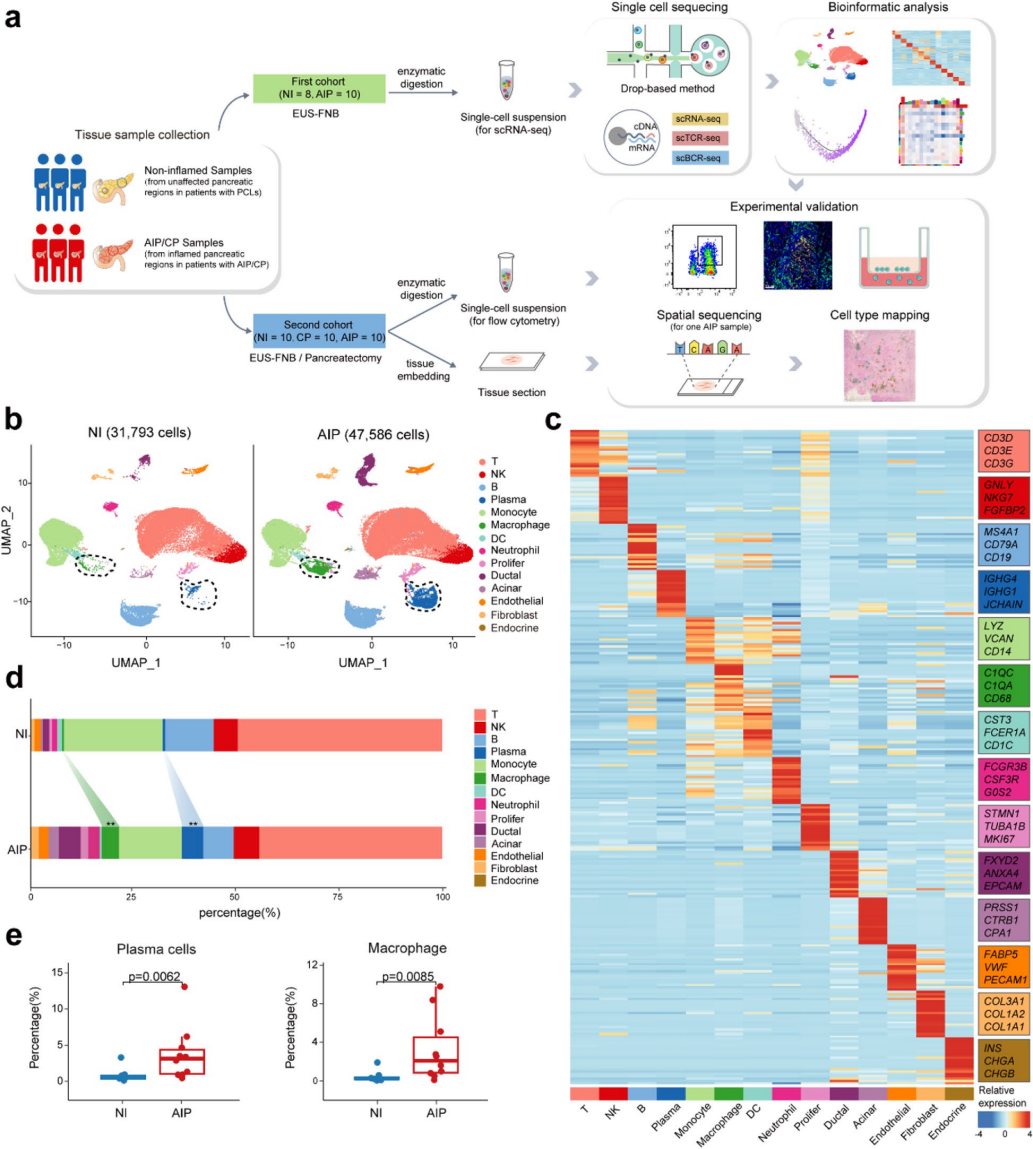
Overview of study design and cellular atlas of pancreatic cells in AIP lesion pancreas. **a** Flowchart depicting the overall experimental design. Lesion and non-inflamed pancreatic tissues were obtained from AIP and NI groups. In the first cohort, samples obtained by EUS-FNA/B were processed into single-cell suspensions and subjected to scRNA-seq, scBCR-seq, and scTCR-seq using 10X Genomics. Single-cell transcriptome data were analysed in detail. Experimental validation and spatial transcriptomic were performed on the second cohort to validate the single-cell analysis findings. **b** UMAP plots showing 14 major cell clusters from non-inflamed (*n* = 8) and AIP (*n* = 10) pancreatic tissues in different colour. Macrophages and plasma cells with significant quantitative changes are circled by dotted lines. **c** Heatmap showing the relative expression (*z* score) of marker genes of indicated major cell clusters. Clusters are coloured as in (**b**). Three representative genes of each cluster were listed in the right rectangles and all top ten maker genes are listed in Additional file 1: Table S4. **d** Bar graph showing the percentage of major cell subsets in non-inflamed and AIP pancreatic tissues. Statistical differences were determined by Wilcoxon rank-sum test (**p < 0.01). **e** Boxplots showing the comparison of the percentage of plasma cells and macrophage in AIP and non-inflamed pancreatic tissues. Statistical differences were determined by Wilcoxon rank-sum test

To explore the cellular transcriptional characteristics of the pancreatic microenvironment in AIP, we analysed pancreatic tissue samples from 10 AIP patients and adjacent normal non-inflamed tissues from 8 PCLs patients as controls, obtained via EUS-FNA/B. There was a disparity in gender distribution between the two groups, as AIP primarily affects males. scRNA-seq and single-cell T cell receptor and B cell receptor sequencing (scTCR/BCR-seq) were performed on a 10X Genomics platform (Fig. 1a). A total of 79,379 high-quality cells were retained after quality control and doublets removal. Unsupervised clustering identified 14 distinct cell clusters, visualized through uniform manifold approximation and projection (UMAP) embeddings (Fig. 1b and Additional file 1: Fig. S1a). These clusters included T-cells, NK cells, proliferating cells, B-cells, plasma cells, myeloid cells (monocytes, macrophages, dendritic cells, and neutrophils), acinar cells, ductal cells, stromal cells (fibroblasts and endothelial cells), and endocrine cells, categorized based on classical markers (Fig. 1c and Additional file 1: Fig. S1b). We observed a notable increase in the proportions of plasma cells (*P* = 0.0062) and macrophages (*P* = 0.0085) in AIP tissues (Fig. 1d,e and Additional file 1: Fig. S1c-d), indicating an activated immune response in the AIP pancreas. Thus, our scRNA-seq analysis provided insights into the altered cellular composition in the pancreatic tissues of AIP patients.

### Increased IgD^−^ ABCs differentiated into plasma cells secreting IgG in AIP lesion pancreatic tissues

**Fig. 2:**
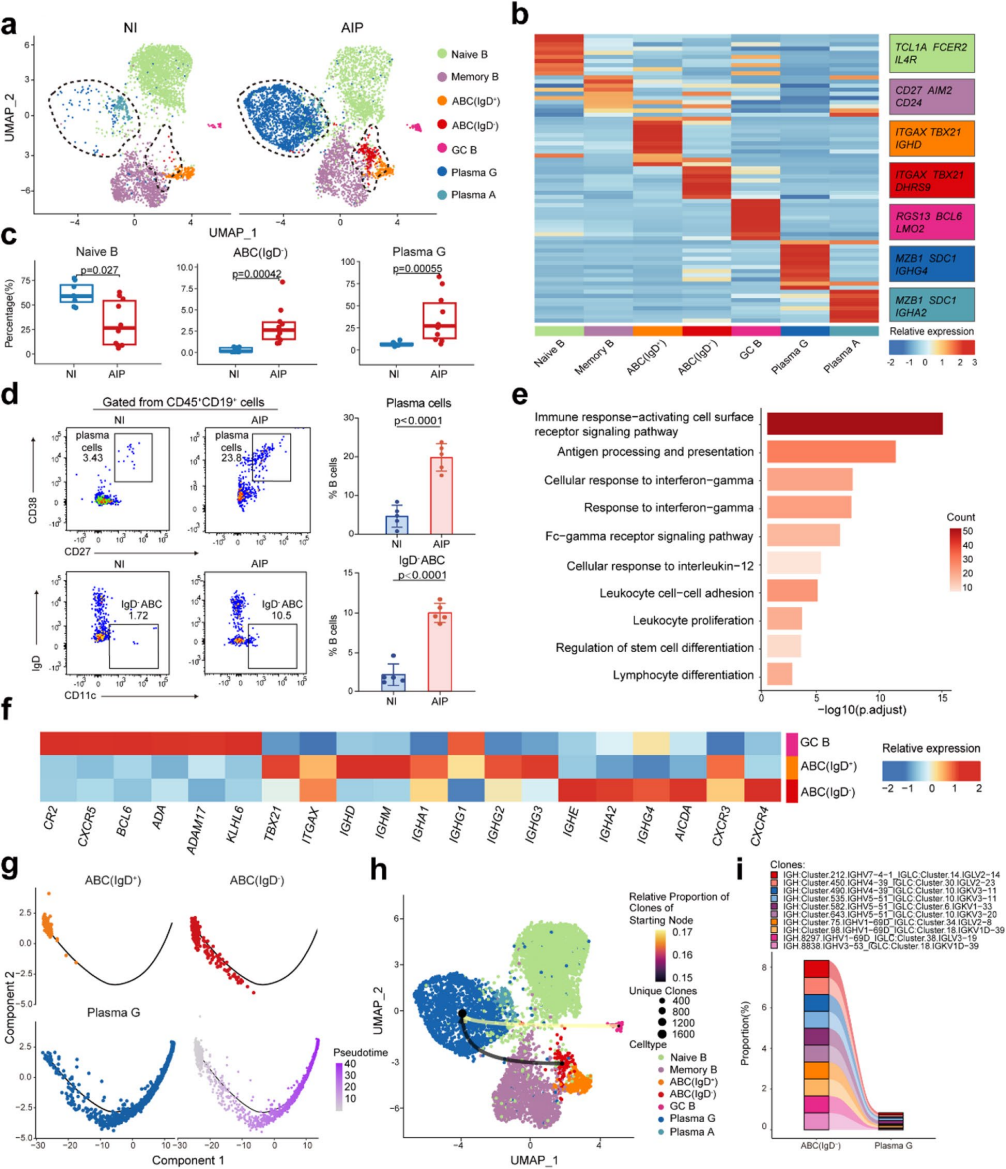
Identification of disease-specific IgD^*−*^ ABCs and their clonal expansion in AIP. **a** UMAP plots showing 7 B-cell subsets in non-inflamed and AIP lesion pancreatic tissues in different colour. **b** Heatmap showing the relative expression (*z* score) of marker genes in each B cell subset. All top ten maker genes are listed in Additional file 1: Table S5. **c** Box graph comparing the percentage of naive B cells, IgD^−^ ABCs and plasma G cells clusters in non-inflamed and AIP pancreatic tissues. Statistical differences were determined by Wilcoxon rank-sum test. **d** Representative flow cytometry plots revealed the proportion variation in IgD^−^ ABCs and plasma cells to B cells in non-inflamed pancreas and AIP pancreatic lesions (*n* = 5). Statistical differences were determined by two-tailed *t*-test. **e** Representative enriched GO biological process terms of differentially expressed genes (DEGs) expressed in IgD^−^ ABCs. A hypergeometric test was performed with false discovery rate (FDR) adjusted *P* values. **f** Heatmap showing the relative expression (*z* score) of germinal centre associated genes and immunoglobulin genes in GC B, IgD^−^ ABCs, and IgD^+^ ABCs. Clusters are coloured as in (**a**). **g** Inferred differentiation trajectory for IgD^+^ ABCs (left top), IgD^−^ ABCs (right top), and plasma G cells (left bottom) by monocle. Each dot represents a cell. Estimated pseudotime was in the right bottom. **h** Weighted and oriented network plot combined with UMAP plots showing the clonotype connection among IgD^−^ ABCs, GC B, and plasma G cells. Dot size represents the number of unique clones. Lines with an arrow point from the starting cluster to the end cluster, and the line colour represents the proportion of shared clonotypes in the starting cluster. **i** Ribbon chart illustrating the proportion of ten shared clones in IgD^−^ ABCs and plasma G cells. Ribbon connects the same clones. Colour of bar and ribbon represents different clones

We further analysed B-cells and plasma cells, identifying seven distinct subsets: naïve B-cells, memory B-cells, IgD^+^ and IgD^−^ ABCs, germinal centre (GC) B-cells, plasma A cells (secreting IgA), and plasma G cells (secreting IgG) (Fig. 2a and Additional file 1: Fig. S2a). These subsets were characterized using specific marker genes for each cluster (Fig. 2b and Additional file 1: Fig. S2b). In the AIP pancreas, plasma G cells, which express high levels of IGHG4, were found to be more prevalent (*P* = 0.00055), whereas naïve B-cells were more common in non-inflamed pancreatic tissues (*P* = 0.027). This indicates a heightened B-cell activation in AIP (Fig. 2c and Additional file 1: Fig. S2c-e). Moreover, we identified ABCs in the human pancreas based on the expression of *ITGAX* (encoding CD11c) and *TBX21* (encoding T-bet), consistent with previous studies [29, 30]. We subclassified these ABCs into two types based on IGHD expression levels (Fig. 2b). Notably, only IgD^−^ ABCs were significantly increased in the AIP pancreas (*P* = 0.00042, Fig. 2c and Additional file 1: Fig. S2c-e). To validate these findings, we conducted flow cytometry on fresh pancreatic tissues from AIP patients obtained via EUS-FNA/B (Additional file 1: Fig. S2f). Consistently, the flow cytometry results confirmed a significant increase in the proportions of plasma G cells and IgD^−^ ABCs in AIP pancreatic tissues (*P* < 0.0001, Fig. 2d).

To further investigate the function of IgD^−^ ABCs, Gene Ontology (GO) enrichment analysis was conducted. This analysis revealed that IgD^−^ ABCs were primarily involved in activating surface receptor signalling pathways, responding to IFN-γ, and processes related to proliferation and differentiation. This suggested that these cells were activated by costimulatory signals from other receptors or cytokines (Fig. 2e). We then investigated whether IgD^−^ ABCs participate in the GC process, where B-cells mature into plasma cells. Comparing GC-associated genes and immunoglobulin heavy chain expression between ABCs and GC B-cells, we found that IgD^−^ ABCs lacked GC markers like CXCR5 and BCL6 but exhibited high levels of AICDA, which encodes activation-induced cytidine deaminase (AID)—an enzyme crucial for B-cell class switching, recombination, and somatic hypermutation [31] (Fig. 2f). Additionally, while GC B-cells primarily expressed IgG1, IgD^−^ ABCs were found to produce IgG4 and IgE isotypes (Fig. 2f). This indicated that IgD^−^ ABCs may have undergone class switching and are likely progressing through a maturation process that is independent of GC involvement.

To investigate the development and differentiation of IgD^−^ ABCs, we conducted pseudotime trajectory analysis. This analysis suggested that IgD^−^ ABCs were predicted to originate from IgD^+^ ABCs and subsequently differentiate into plasma G cells (Fig. 2g). This progression was marked by a downregulation of genes associated with early B-cell development and an upregulation of immunoglobulin genes (Additional file 1: Fig. S2g). We further analysed the isotype usage and clonal composition of B-cell subsets using scBCR-seq. Our findings showed that all four IgG subclasses were expanded in AIP pancreatic tissues (Additional file 1: Fig. S3a-b). Clonotype analysis indicated a decrease in clonal diversity (ACE, *P* = 0.011; chao1, *P* = 0.0079; gini.simpson, *P* = 0.0037; inv.simpson, *P* = 0.0037; norm.entropy, *P* = 0.0037; Shannon, *P* = 0.0037) and a reduction of unique clones (*P* = 0.0025) in AIP lesions, suggesting an expansion of specific clonotypes (Additional file 1: Fig. S3c-d). Plasma cells, GC B-cells, and IgD^−^ ABCs exhibited higher proportions of medium or large clones compared to other B-cell subtypes (Additional file 1: Fig. S3e-f). Integrated scRNA-seq and scBCR-seq analyses revealed that plasma G cells shared some clonotypes with IgD^−^ ABCs, although GC B-cells showed the highest degree of clonotype overlap with plasma G cells (Fig. 2h and Additional file 1: Fig. S3g). Notably, we identified 10 specific clonotypes in IgD^−^ ABCs that were also present in plasma G cells in our data (Fig. 2i). These results suggested that IgD^−^ ABCs in AIP patients can differentiate into immunoglobulin-producing plasma G cells, indicating their role in disease-specific immune responses.

### CXCL9^+^ macrophages promoted the migration of IgD^−^ ABCs in AIP via CXCL9-CXCR3 axis

**Fig. 3:**
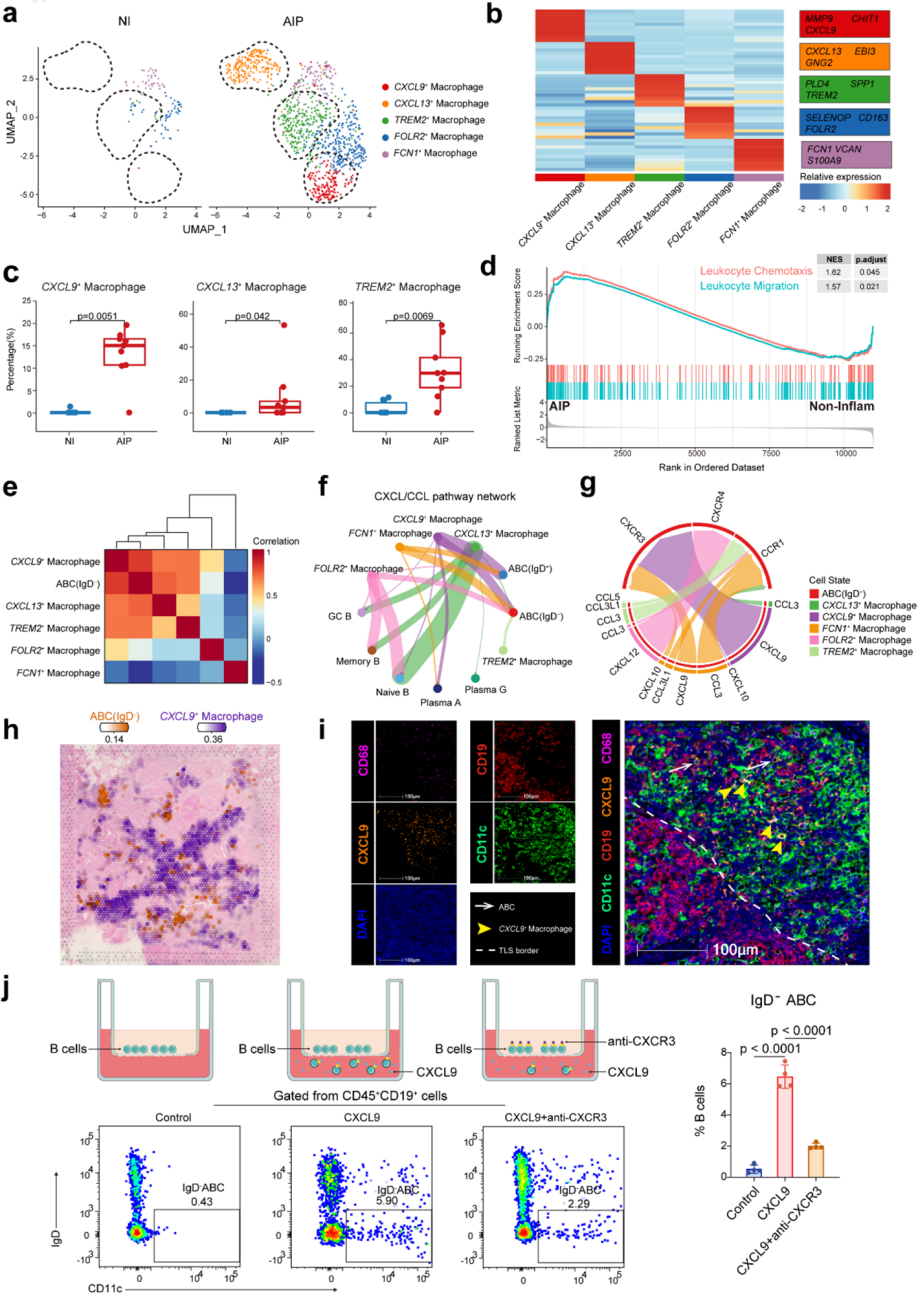
*CXCL9*^+^ macrophages promoted the migration of IgD^*−*^ ABCs in AIP via CXCL9-CXCR3 axis. **a** UMAP plots showing 5 macrophage subsets in non-inflamed and AIP lesion pancreatic tissues in different colour. **b** Heatmap showing the relative expression (*z* score) of marker genes in subclustered macrophages. All top ten maker genes are listed in Additional file 1: Table S6. **c** Box graph comparing the percentage of *CXCL9*^+^ macrophages,* CXCL13*^+^ macrophages, and *TREM2*^+^ macrophages in non-inflamed and AIP pancreatic tissues. Statistical differences were determined by Wilcoxon rank-sum test. **d** GSEA enrichment analysis of leukocyte chemotaxis and leukocyte migration gene sets from C5: ontology gene sets in MSigDB database between macrophages from AIP group and non-inflamed group. NES, normalization enrichment score. **e** Heatmap showing the spearman correlation between the percentages of IgD^*−*^ ABCs and macrophage subsets across 18 scRNA-seq samples. **f** Circle plot displaying the interactions of CXCL/CCL pathway network from macrophages subsets to B-cell subsets. The width of edges represents the strength of the communication. **g** Chord plot displaying the ligand–receptor interactions of CXCL/CCL pathway network from macrophages subsets to IgD^*−*^ ABCs. The width of edges represents the strength of the communication. **h** Spatial transcriptomics showing the spatial distribution of IgD^*−*^ ABCs and *CXCL9*^+^ macrophages. **i** Representative multi-colour immunofluorescence (mIF) staining of ABCs and *CXCL9*^+^ macrophages in human AIP lesion pancreatic tissue (× 20). DAPI (blue), CD19 (red), CD11c (green), CXCL9 (orange), and CD68 (purple) in merged channels are shown. Bar, 100 μm. TLS boundaries are outlined by white dashed lines (TLS: lower left quadrant). White/yellow arrows denote ABCs and *CXCL9*^+^ macrophages respectively. **j** Diagrams and representative flow cytometry plots of IgD^*−*^ ABCs that migrated to the lower chamber in the chemotaxis assays. Flow cytometry analysis revealed the proportion variation of migrated IgD^*−*^ ABCs in controls, CXCL9 and CXCL9 + anti-CXCR3 (*n* = 4). Statistical differences were determined by two-tailed *t*-test

The evolution of scRNA-seq and spatially resolved transcriptomics has unveiled unprecedented insights into the dynamic heterogeneity of macrophage subsets across pathological conditions in digestive organs [32, 33]. As expected, macrophages were notably abundant in the pancreatic lesions of AIP patients, a finding confirmed by flow cytometry (*P* = 0.0475, Fig. 1e and Additional file 1: Fig. S4a). To further understand the role of macrophages in AIP, we subclustered them into five subsets based on marker genes (Fig. 3a,b and Additional file 1: Fig. S4b-c). We observed a significant increase in *CXCL9*^+^ (*P* = 0.0051) and *CXCL13*^+^ macrophages (*P* = 0.042), which expressed higher levels of chemoattractant molecules, as well as *TREM2*^+^ macrophages (*P* = 0.0069) in AIP pancreatic tissues (Fig. 3c and Additional file 1: Fig. S4d-f). Remarkably, GSEA analysis revealed that macrophages in the AIP pancreas exhibited enhanced function of leukocyte chemotaxis and migration (Fig. 3d). Given the elevated levels of both IgD^−^ ABCs and macrophages, we investigated which macrophage subset might recruit IgD^−^ ABCs into the pancreas. Our analysis showed a strong correlation between the abundance of *CXCL9*^+^ macrophages and IgD^−^ ABCs (Fig. 3e). We also examined interactions of CXCL and CCL signalling pathways between macrophages and B-cell subsets. *CXCL9*^+^ macrophages exhibited the strongest chemotactic interaction with IgD^−^ ABCs, with the CXCL9-CXCR3 receptor-ligand pair being the most dominant (Fig. 3f,g). This indicated that *CXCL9*^+^ macrophages played a central role in attracting CXCR3-expressing IgD^−^ ABCs. By analysing the spatial transcriptome sequencing data of a representative AIP pancreatic region, we observed that *CXCL9*^+^ macrophages were diffusely distributed, primarily localized at the periphery or more distant regions of TLS, rather than within the TLS core. At the TLS periphery, these macrophages exhibited proximal positioning to IgD^−^ABCs (Fig. 3h). Their spatial distribution supports the interactions between the two cell populations identified in our single-cell data. To further investigate the specific regions where *CXCL9*^+^ macrophages and ABCs colocalize, we performed multiplex immunofluorescence. Through this analysis, we observed that *CXCL9*^+^ macrophages and ABCs were colocalized at the peripheral regions of TLS in patients with AIP (Fig. 3i). To validate our findings, we conducted a CXCL9-mediated transwell chemotaxis assay. Peripheral B-cells from AIP patients were placed in the upper chamber and allowed to migrate towards the lower chamber, which contained either human recombinant CXCL9 or no chemotactic factor. We observed a significantly higher percentage of IgD^−^ ABCs in the lower chamber with CXCL9 compared to the control, and this migration was partially inhibited by CXCR3-blocking antibodies (*P* < 0.0001, Fig. 3j). To more directly investigate the relationship at the cellular level, we repeated the chemotaxis assay using M1-polarized macrophages derived from THP-1 cells with CXCL9 knockdown by siRNA. Knockdown efficiency was confirmed prior to functional assessment (*P* = 0.0011). CXCL9-deficient macrophages exhibited reduced chemoattraction towards IgD^*−*^ ABCs from AIP patients versus negative siRNA controls (*P* = 0.0065, Additional file 1: Fig. 4Sg). These results confirmed that the CXCL9/CXCR3 axis was crucial for the migration of IgD^*−*^ ABCs in AIP patients.

**Fig. 4:**
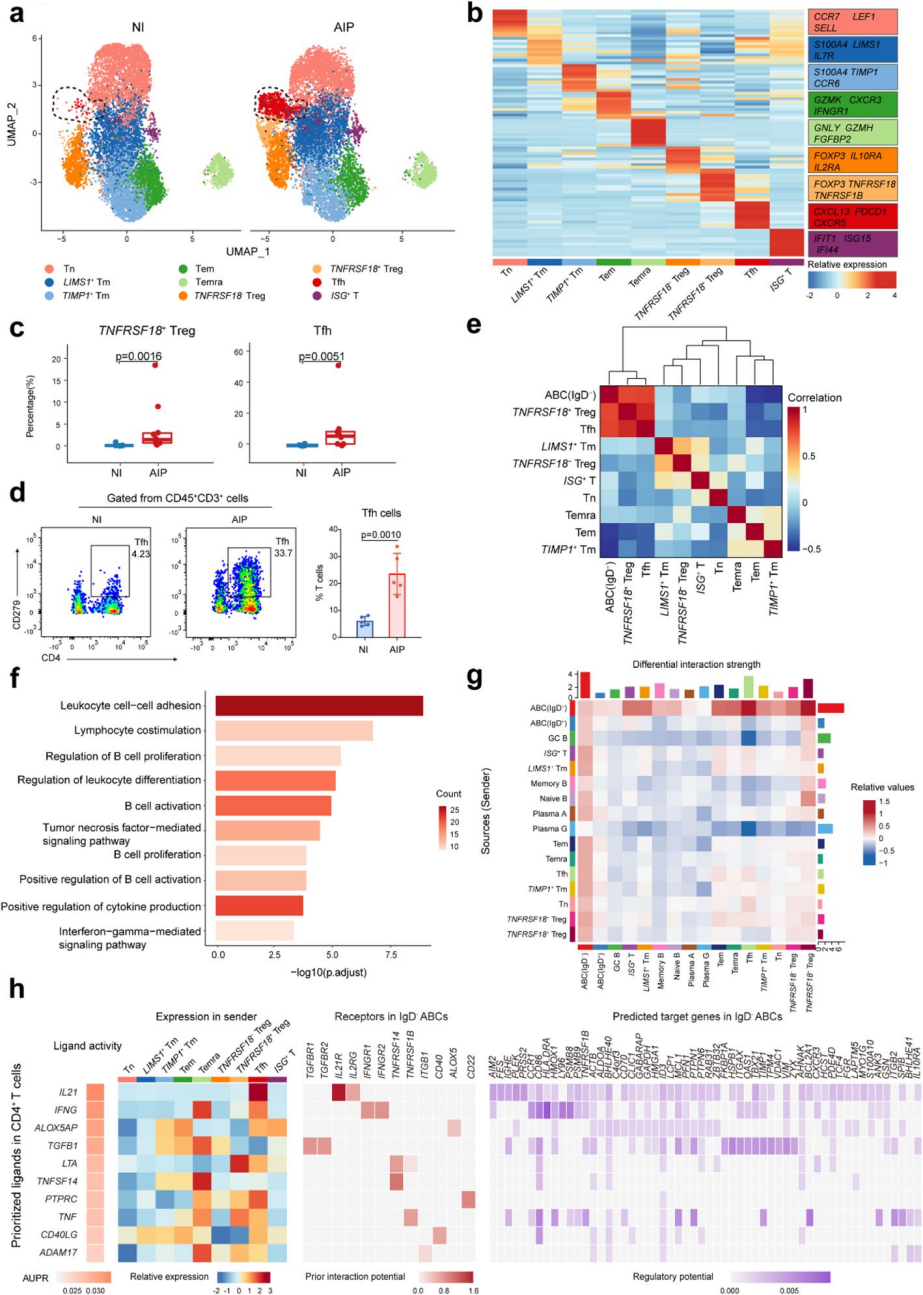
Tfhs expanded in AIP pancreatic tissue and interacted with IgD^*−*^ ABCs via IL-21. **a** UMAP plots showing 9 *CD4*^+^ T-cell subsets in non-inflamed and AIP lesion pancreatic tissues in different colour. **b** Heatmap showing the relative expression (*z* score) of marker genes in subclustered *CD4*^+^ T cells. All top ten maker genes were listed in Additional file 1: Table S7. **c** Box graph comparing the percentage of *TNFRSF18*^+^ Tregs and Tfhs in non-inflamed and AIP pancreatic tissues. Statistical differences were determined by Wilcoxon rank-sum test. **d** Representative flow cytometry plots of Tfhs in non-inflamed pancreas and AIP pancreatic lesions. Flow cytometry analysis revealed the proportion variation in Tfhs to CD3^+^ T cells in non-inflamed pancreas and AIP pancreatic lesions. Points representing controls (*n* = 5) and AIP pancreatic samples (*n* = 5). Statistical differences were determined by two-tailed *t*-test. **e** Heatmap showing the spearman correlation between the percentages of IgD^*−*^ ABCs and CD4^+^ T-cells subsets across 18 scRNA-seq samples. **f** Representative enriched GO biological process terms of DEGs expressed in Tfhs. A hypergeometric test was performed with FDR adjusted *P* values. **g** Heatmap showing the differential interaction strength among all *CD4*^+^ T-cell subsets and B-cell subsets. **h** Heatmap showing the activity of the top-ranked ligands inferred to regulate IgD^*−*^ ABCs by *CD4*^+^ T-cells according to NicheNet (left), relative expression of top-ranked ligands in each *CD4*^+^ T-cell subsets (middle left), the ligand‒receptor interaction between *CD4*^+^ T-cells and IgD^*−*^ ABCs ordered by ligand activity (middle right), and the downstream target genes in IgD^*−*^ ABCs regulated by the top-ranked ligands (right). AUPR, Area Under the Precision-Recall Curve

### Tfhs expanded in AIP pancreatic tissue and interacted with IgD^−^ ABCs via IL-21

To investigate the role of T-cells in AIP, we performed a subclustered analysis (Additional file 1: Fig. S5a-b). Focusing on CD4^+^ T-cells, we identified 9 distinct subsets based on canonical CD4^+^ T-cell markers (Fig. 4a,b and Additional file 1: Fig. S5c-d). Notably, the proportions of *TNFRSF18*^+^ regulatory T-cells (Tregs, *P* = 0.0016) and Tfhs (*P* = 0.0051) were significantly higher in AIP pancreatic tissues (Fig. 4c and Additional file 1: Fig. S5e-g). This increase in Tfhs was further confirmed by flow cytometry (*P* = 0.0010, Fig. 4d).

Given the observed positive correlation between the abundance of Tfhs and IgD^*−*^ ABCs in AIP patients (Fig. 4e), we aimed to elucidate the potential crosstalk between them within pancreatic lesions. GO enrichment analysis indicated that Tfhs were involved in processes related to cell‒cell adhesion, B-cell proliferation, and activation (Fig. 4f), suggesting that Tfhs played a role in activating B-cells. Cellular interaction analysis using CellChat revealed that IgD^*−*^ ABCs interacted more strongly with CD4^+^ T-cells compared to other B-cell subsets (Fig. 4g). Further NicheNet analysis of interactions between CD4^+^ T-cells and IgD^*−*^ ABCs highlighted IL-21 as a key ligand. IL-21, highly expressed in Tfhs, binds to its receptors IL21R/IL2RG on IgD^*−*^ ABCs, leading to the expression of genes associated with B-cell differentiation and activation (Fig. 4h). Additionally, ligand-receptor pairs involved in cellular adhesion, such as ICAM2-ITGAM/ITGB2 and CD40LG-CD40, may also facilitate the interaction between Tfhs and IgD^*−*^ ABCs (Additional file 1: Fig. S5h).

### Tfhs-ABC interactions at the periphery of TLS

**Fig. 5:**
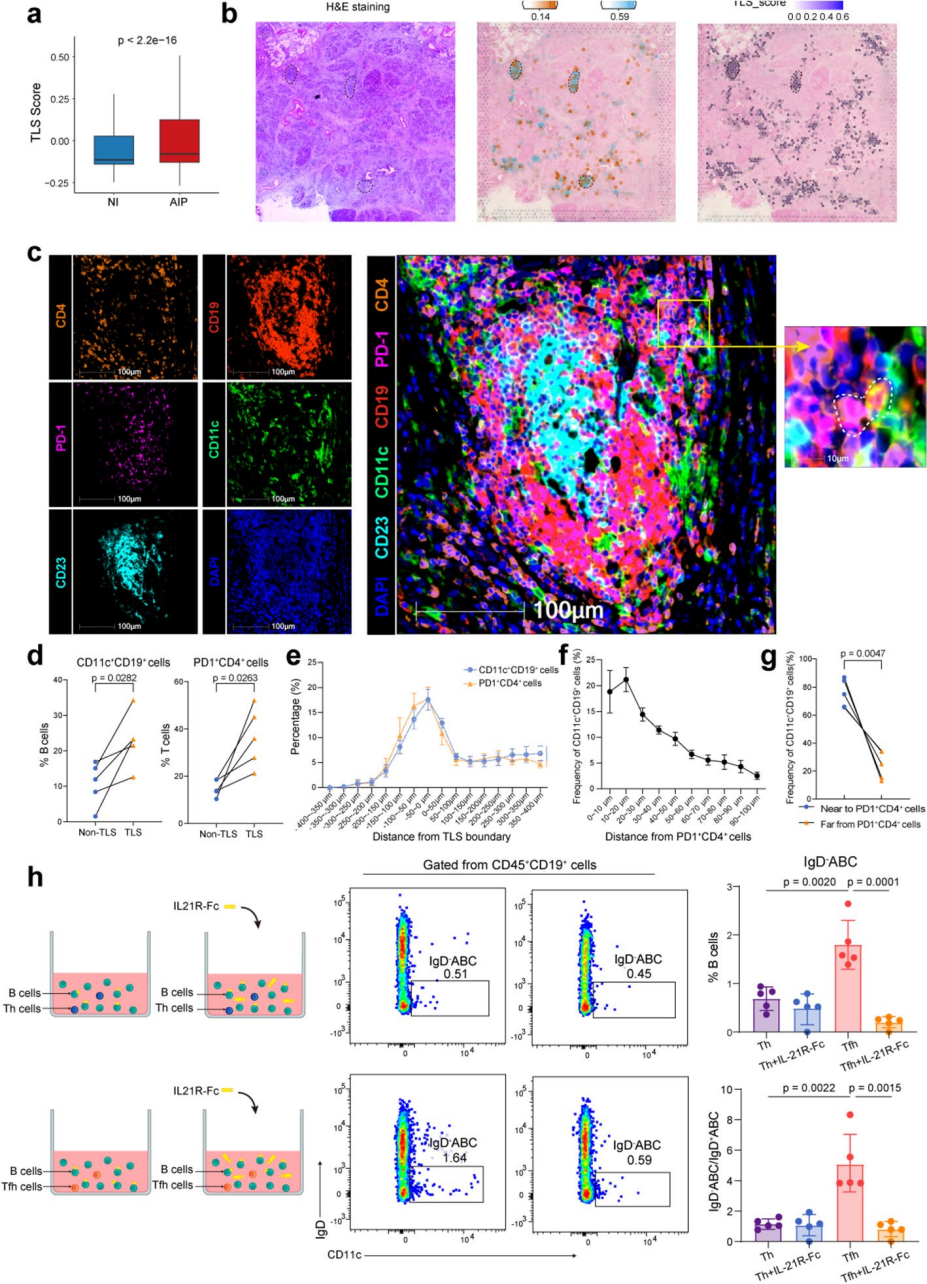
Tfhs-ABC interactions at the periphery of TLSs. **a** Boxplot comparing the TLS signature score of all cells in non-inflamed and AIP lesion pancreatic tissues (two-tailed *t*-test). **b** Spatial transcriptomics slide of pancreatic tissue from a representative AIP patient. H&E staining (left), spatial distribution of IgD^*−*^ ABCs and Tfhs (middle), and TLS signature score (right). Typical TLS structures are circled by dotted lines. **c** Representative mIF staining of TLS in human AIP lesion pancreatic tissue (× 20). DAPI (blue), CD19 (red), CD11c (green), CD23 (cyan), CD4 (orange), and PD1 (purple) in merged channels are shown. Bar, 100 μm. The dotted line circled out the close colocalization between CD19^+^CD11c^+^ ABCs and CD4^+^PD1^+^ Tfhs in a high-power field (right). **d** Comparison of percentages of ABCs to CD19^+^ cells and Tfhs to CD4^+^ cells in TLS or non-TLS area from paired AIP pancreatic tissue slides (*n* = 5, paired two-tailed *t*-test). **e** The percentage distribution of ABCs and Tfhs within serial bands of the distances from these cells to TLS boundary. Negative values represent the inside of the TLS and positive values represent the outside of the TLS. (*n* = 5, mean ± SEM). **f** The percentage distribution of ABCs within serial bands of the distances to Tfhs (*n* = 5, mean ± SEM). **g** Comparison of the proportion of ABCs near to Tfhs (< 50 μm) and far from Tfhs (> 50 μm) in AIP lesion pancreatic tissues (*n* = 5, paired two-tailed *t*-test). **h** Schematic diagram (left) and representative flow cytometry plots (middle) showing T-B cell co-culture assays. Circulating Tfhs and Th cells from AIP patients were cocultured with isolated healthy blood B cells in the presence or absence of isotype and anti-IL-21R. Frequency of IgD^*−*^ ABCs among B cells (upper right) and the IgD^*−*^/IgD.^+^ ABCs ratio (lower right) across 4 groups are shown (mean ± SD, two-tailed *t*-test)

Given that Tfhs and B-cells often interact within TLSs to support B-cell maturation, we investigated the spatial architecture and relationship among Tfhs, ABCs, and TLSs in AIP pancreatic tissue. First, our analysis revealed that AIP pancreatic specimens exhibited significantly higher TLS gene signature scores compared to non-inflamed controls (*P* < 2.2e − 16, Fig. 5a). H&E staining confirmed the presence of TLSs in AIP pancreatic tissue. Furthermore, combined with ST of a representative AIP pancreatic region and scRNA-seq data, we observed a strong colocalization of Tfhs and IgD^*−*^ ABCs in regions with elevated TLS scores, indicating their interaction within TLSs in AIP pancreases (Fig. 5b).

Six-colour immunofluorescence staining further confirmed TLSs in AIP pancreatic tissues. These TLSs were characterized by a central core of dense CD19^+^ B-cell and CD23^+^ follicular dendritic cell clusters, surrounded by a peripheral layer of ABCs (CD19^+^CD11c^+^) and Tfhs (CD4^+^PD1^+^) (Fig. 5c). Using HALO, a quantitative image analysis tool, we found that both ABCs (*P* = 0.0282) and Tfhs (*P* = 0.0263) were more abundant in TLS regions compared to non-TLS regions (Fig. 5d). Infiltration analysis showed that ABCs and Tfhs were primarily located near the TLS boundary (− 100 ~ 0 μm), rather than at the TLS centre (Fig. 5e). Immunofluorescence staining revealed the spatial proximity of ABCs and Tfhs at the TLS periphery (Fig. 5c). Quantitative spatial distance analysis also demonstrated the close spatial relationship between these cell types (*P* = 0.0047, Fig. 5f,g). These findings highlight the interaction between Tfhs and ABCs at the periphery of TLSs, suggesting that ABCs undergo GC-independent development. To further validate the interaction between Tfhs and ABCs, we sorted circulating Tfhs and Ths by FACS from AIP patients and coculture them with isolated healthy blood B cells in the presence of isotype or anti-IL-21R (Additional file 1: Fig. S6a). Flow cytometry revealed that Tfhs significantly enhanced IgD^*−*^ ABC differentiation compared to Th cells (*P* = 0.0020, Fig. 5h). Parallel conditions with/without IL-21R-Fc chimaera blockade demonstrated that Tfh enhancement of IgD^*−*^ ABC differentiation was depended on IL-21 signalling (*P* = 0.0001, Fig. 5h). Collectively, these results provide compelling evidence for functional interactions between Tfhs and ABCs.

### CD8^+^ T-cells and the clonality of T-cells in the pancreas of AIP patients

We further analysed CD8^+^ T-cells by clustering them to examine changes during AIP development. We identified 5 distinct clusters based on their marker genes (Additional file 1: Fig. S7a-b). Notably, only tissue-resident memory T (Trm) cells were significantly enriched in the pancreas of AIP patients (*P* = 0.023, Additional file 1: Fig. S7c). GO analysis revealed that Trm cells were involved in T-cell activation, differentiation, cell chemotaxis, and IFN-γ production (Additional file 1: Fig. S7d). Trm cells also exhibited the strongest interactions with IgD^*−*^ ABCs among all CD8^+^ T-cell subsets (Additional file 1: Fig. S7e). Additionally, the ligand-receptor pairs associated with Trm cells, such as MIF-CD74 and IFNG-IFNGR, are known to contribute to autoimmune disease pathogenesis [34, 35] (Additional file 1: Fig. S7f). These findings suggested that Trm cells may play a role in the development and progression of AIP.

To examine T-cell clonality in non-inflamed and AIP pancreatic tissues, we conducted integrated scRNA-seq and scTCR-seq analyses. These analyses showed no significant difference in clonal diversity or unique clones between the two groups (Additional file 1: Fig. S8a-b). Among the T-cell subsets, terminally differentiated effector memory T cells (Temra) were the most expanded in AIP tissues (Additional file 1: Fig. S8c-d). TCR clonal overlap analysis indicated moderate clonal sharing between Temra and effector memory T cells (Tem), suggesting close lineage relationships between these subsets (Additional file: Fig. S8e).

### Unique immunological profiles in the microenvironments of AIP versus CP

**Fig. 6:**
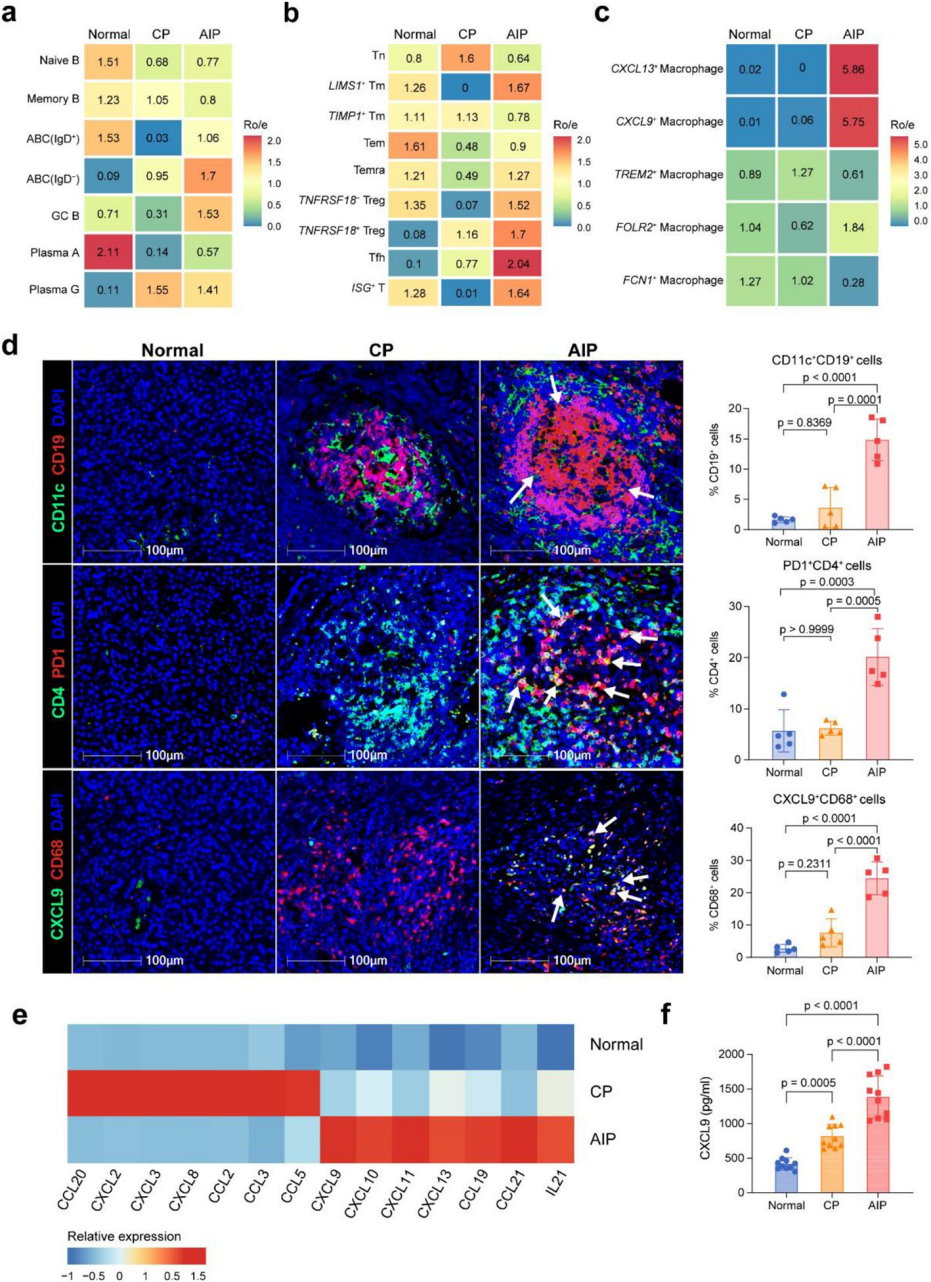
Unique immunological profiles in the microenvironments of AIP versus CP. **a** Heatmap showing the preference of B cell subsets among AIP, CP, and NI estimated by Ro/e score. Colour represent Ro/e index. **b** Heatmap showing the preference of CD4^+^ T cell subsets among AIP, CP, and NI estimated by Ro/e score. Colour represent Ro/e index. **c** Heatmap showing the preference of macrophage subsets among AIP, CP, and NI estimated by Ro/e score. Colour represent Ro/e index. **d** Representative immunofluorescence staining of ABCs (top), Tfhs (medium), and *CXCL9*^+^ macrophages (bottom) and quantitative analysis (right) from AIP, CP, and NI groups (× 20, 5 patients per group). DAPI (blue), CD19 (red), CD11c (green), PD1 (red) CD4 (green), CD68 (red), and CXCL9 (green) in merged channels are shown respectively. Bar, 100 μm. Statistical differences were determined by the one-way ANOVA with Bonferroni correction. Data represent the mean ± SD. **e** Heatmap showing the relative expression (*z* score) of selective genes in AIP, CP, and NI groups. **f** Bar plots showing the plasma concentration (pg/ml) of CXCL9 from AIP, CP, and NI groups (10 patients per group). Data represent the mean ± SD. Statistical differences were determined by the one-way ANOVA with Bonferroni correction

To assess the specificity of transcriptomic signatures in AIP, we integrated our scRNA-seq data with that from Bomi Lee’s research on CP tissues (Additional file 1: Fig. S9a-c) [15]. By comparing the pancreatic immune landscapes between AIP and CP, and calculating the ratio of observed over expected cell numbers (Ro/e), we identified a significant enrichment of IgD^*−*^ ABCs, Tfhs, and *CXCL9*^+^ macrophages in AIP tissues compared to CP, underscoring their unique contribution to the AIP microenvironment (Fig. 6a–c and Additional file 1: Fig. S9d-f). This was further validated by immunofluorescence staining, which confirmed elevated levels of ABCs (*P* = 0.0001), Tfhs (*P* = 0.0005), and *CXCL9*^+^ macrophages (*P* < 0.0001) in AIP pancreatic tissues (Fig. 6d).

Our analysis of chemokine expression patterns revealed notable differences: CCL20, CXCL2, and CXCL3 were upregulated in CP, while CXCL9, CXCL10, and CXCL11 (ligands for CXCR3) were enriched in AIP. This suggests that CXCR3-mediated immune responses play a crucial role in AIP. Additionally, cytokines associated with B-cell expansion, such as IL-21, and those involved in TLS formation, including CCL19, CCL21, and CXCL13, were also elevated in AIP pancreatic tissues, indicating enhanced T-B cell interactions (Fig. 6e). ELISA further confirmed increased plasma levels of CXCL9 in AIP patients compared to those with CP and control groups (*P* < 0.0001, Fig. 6f), suggesting its potential as a biomarker for AIP. These findings highlight the distinct immune profiles of AIP and CP, emphasizing the potential of these immune markers as targets for future research and therapeutic strategies in AIP.

## Discussion

Research into IgG4-related sialadenitis has progressed more rapidly than studies on AIP, primarily due to easier access to sialadenitis samples compared to pancreatic tissues [14, 36–38]. AIP often presents as a pancreatic mass or diffuse enlargement, making it susceptible to misdiagnosis as pancreatic cancer, which can lead to unnecessary surgical treatments. Prior to 2018, in situ studies on human AIP were largely limited to immunophenotyping, based on a small number of surgical specimens [12, 39–42]. As diagnostic technologies have improved, fewer AIP patients undergo surgery, making it challenging to obtain surgical specimens for studying in situ immunity. However, advancements in EUS-FNA/B technologies offer high accuracy and safety in diagnosing pancreatic lesions and provide a minimally invasive method to acquire pancreatic tissue for histological analysis and research [43, 44]. This technique has allowed us to obtain sufficient AIP pancreatic samples to perform multi-omics sequencing, enabling a comprehensive mapping of the cellular landscape within the AIP pancreatic microenvironment.

ABCs, a specialized group of memory B-cells, emerge with age, infection, and autoimmunity. In autoimmune diseases such as systemic lupus erythematosus, ABCs migrate to affected tissues, contributing to disease progression by producing autoantibodies and inflammatory cytokines [29, 30]. A recent study showed that inhibition of the critical transcription factor ZEB2 in ABCs could alleviate lupus pathogenesis, suggesting the therapeutic potential of targeting ABCs in autoimmune disease [30]. Our study is the first to describe the expansion and possible pathogenic role of IgD^*−*^ ABCs in the pancreas of AIP patients. Given ABCs’ role in autoantibody production in other diseases, we hypothesize they may also contribute to autoantibody production in AIP [45]. However, further research is needed to understand the pathogenicity and autoreactivity of antibodies from highly clonally expanded ABCs. The effectiveness of rituximab in relieving AIP/IgG4-RD [46] combined with the higher levels of CD20 expression on ABCs than on other B-cell subsets [47] also indicates that targeting ABCs might be a promising strategy for AIP treatment.

CXCL9, also known as IFN-γ inducible monokine, participates in Th1-predominant immune responses [48]. It has also been reported to attract immune cells, especially T-cells and monocytes, in chronic pancreatitis [49, 50]. In our study, we discovered that *CXCL9*^+^ macrophages can recruit ABCs through the CXCL9/CXCR3 axis, a finding supported by both scRNA-seq data and chemotaxis assays. This suggests that targeting the CXCL9/CXCR3 pathway could offer a new therapeutic approach for AIP. CXCL9 expression in macrophages can be triggered by factors such as IFN-γ, TNF-α, and lipopolysaccharides [50]. We hypothesize that IFN-γ, produced by activated Th1 cells or CD4^+^ (GZMA^+^) cytotoxic T lymphocytes, which are also abundant in AIP/IgG4-RD tissues and exacerbate fibro-inflammatory responses, may be a primary inducer of CXCL9 [14, 51, 52]. However, further research is needed to validate this hypothesis.

Tfhs are linked with IgG4^+^ plasma cells and disease activity in IgG4-RD, with their interactions with B-cells in TLSs previously thought to drive these associations [7, 11, 53–55]. TLSs have been observed in various autoimmune diseases and tumours, contributing to in situ antigen-specific immune responses [56–59]. However, the pancreas has traditionally been considered an immunologically “cold” organ. Consequently, the positive correlation between TLSs and responses to immunotherapy has not been established in PDAC [60, 61]. Interestingly, we observed that TLSs in the pancreas of AIP patients contained both Tfhs and ABCs. Notably, ABCs were primarily located at the periphery of TLSs rather than within them, a pattern also observed in recent studies of infections and tumours, suggesting a GC-independent activation process for ABCs [62, 63]. These findings indicate that immune responses in the AIP pancreas are active and highly regulated. However, the precise cellular and molecular mechanisms driving pancreatic TLS formation and their role in AIP pathogenesis are still not fully understood.

Our study has several limitations. First, the interpretability of our scRNA-seq findings is constrained by inherent limitations in sample size and significant sex disparity. This demographic bias mirrors the well-established epidemiological profile of AIP, a relatively rare condition with an estimated prevalence of 10.1 per 100,000 individuals and annual incidence of 3.1 per 100,000 [64]. The disease predominantly affects elderly males (mean age: 68.1 years; male-to-female ratio: 2.94), as consistently reported in regional epidemiological studies [64]. These intrinsic epidemiological characteristics inevitably impact the external validity of our conclusions, necessitating validation through larger, multicentre cohorts with balanced demographic representation to confirm the robustness of our observations. Second, the fresh tissue samples obtained via EUS-FNA/B might not capture all stromal cell types, possibly missing important cellular subsets. Lastly, many of our conclusions about cellular functions are based on bioinformatics data, requiring further validation through in vivo or in vitro experiments.

## Conclusions

In conclusion, our study utilized multi-omics analysis and validation experiments to reveal the transcriptional characteristics and spatial distribution of distinct cell subsets in the pancreatic microenvironment of AIP patients. These findings enhance our understanding of local immune responses and propose a potential pathogenic model involving ABCs, Tfhs, and macrophages, offering insights for future therapeutic strategies.

## Supplementary Information

Additional file 1: Fig. S1. The landscape of AIP pancreatic microenvironment revealed by single-cell sequencing. Fig. S2. Heterogeneity of the B cells in the pancreas of AIP patients. Fig. S3. BCR analysis of all B cell subsets in the pancreas of AIP. Fig. S4. Heterogeneity of the macrophages in the pancreas of AIP patients. Fig. S5. Heterogeneity of the CD4 + T-cells in the pancreas of AIP patients. Fig. S6. Flow cytometry gating strategy for Th and Tfh cells in co-culture experiments. Fig. S7. Heterogeneity of the CD8 + T-cells in the pancreas of AIP patients. Fig. S8. TCR analysis of all T cell subsets in the pancreas of AIP. Fig. S9. Comparison of the immune cell profile of AIP, CP and NI groups. Table S1. Clinical information of AIP patients and non-inflamed controls included for scRNA-seq. Table S2. Clinical information of AIP patients and non-inflamed controls included for flow cytometry validation. Table S3. Clinical information of AIP and CP patients and non-inflamed controls included for multiplex immunofluorescent. Table S4. Top10 marker genes for main cell types. Table S5. Top 10 marker genes for B cell subclusters. Table S6. Top 10 marker genes for macrophage subclusters. Table S7. Top 10 marker genes for CD4 + T cell subclusters. Table S8. Top 10 marker genes for CD8 + T cell subclusters. Table S9. Signature genes used to define functional gene sets..

## Data Availability

Raw sequencing reads of all scRNA-seq, scTCR/BCR-seq and ST for human AIP samples have been deposited in the Genome Sequence Archive (GSA) and with data accession ID: HRA007090 (https://ngdc.cncb.ac.cn/gsa-human/browse/HRA007090) [65]. scRNA-seq data of CP were acquired from the GEO database at https://www.ncbi.nlm.nih.gov/geo/ in GSE165045 [15, 27]. No new algorithms were developed for this manuscript. All code generated for analysis is available is available at github ( [https://github.com/JiaxinWang6542/AIP](https:/github.com/JiaxinWang6542/AIP) ) [66] and Zenodo (https://zenodo.org/records/17226920) [67].

## Abbreviations

ABCs: Age-associated B cells
AID: Activation-induced cytidine deaminase
AIP: Type 1 autoimmune pancreatitis
ANOVA: Analysis of variance
CP: Chronic pancreatitis
DEGs: Differentially expressed genes
ELISA: Enzyme-Linked Immunosorbent Assay
EUS: Endoscopic ultrasound
EUS-FNA/B: Endoscopic ultrasound-guided fine-needle aspiration/biopsy
EDTA: Ethylenediaminetetraacetic acid
FACS: Fluorescence-activated cell sorting
FBS: Foetal bovine serum
FDR: False discovery rate
FFPE: Formalin-fixed paraffin-embedded
GC: Germinal centre
GEO: Gene Expression Omnibus
GO BP: Gene Ontology biological process
GO: Gene Ontology
GSA: Genome Sequence Archive
GSEA: Gene Set Enrichment Analysis
H&E: Haematoxylin and eosin
ICDC: International Consensus Diagnostic Criteria
IFN-γ: Interferon-gamma
IgG4-RD: IgG4-related disease
LPS: Lipopolysaccharides
mIF: Multi-colour immunofluorescence
MSigDB: The Molecular Signatures Database
NES: Normalization enrichment score
NI: Non-inflamed control
PBS: Phosphate-buffered saline
PCA: Principal component analysis
PCLs: Pancreas cystic lesions
PDAC: Pancreatic ductal adenocarcinoma
PMA: Phorbol ester
Ro/e: The ratio of observed over expected cell numbers
RT-qPCR: Reverse transcription-quantitative polymerase chain reaction
scRNA-seq: Single-cell RNA sequencing
scTCR/BCR-seq: Single-cell immune receptor repertoire sequencing
siRNA: Small interfering RNA
ST: Spatial transcriptomics
TBIL: Total bilirubin
Tem: Effector memory T cells
Temra: Terminally differentiated effector memory T cells
Tfhs: T follicular helper cells
Ths: T helper cells
TLSs: Tertiary lymphoid structures
Tregs: Regulatory T-cells
Trm: Tissue-resident memory T cells
TSA: Tyramide signal amplification
UMAP: Uniform manifold approximation and projection
y/o: Years old
