# Supplementary material for: Single-cell multi-omics analysis revealed the expansion of age-associated B cells in the pancreas of type 1 autoimmune pancreatitis patients
Source: Genome Med. 2025 Nov 4;17:138. doi: 10.1186/s13073-025-01567-w (PMC12584476; doi:10.1186/s13073-025-01567-w)
Supplement: Supplementary file 1 — Additional file 1: Fig. S1. The landscape of AIP pancreatic microenvironment revealed by single-cell sequencing. Fig. S2. Heterogeneity of the B cells in the pancreas of AIP patients. Fig. S3. BCR analysis of all B cell subsets in the pancreas of AIP. Fig. S4. Heterogeneity of the macrophages in the pancreas of AIP patients. Fig. S5. Heterogeneity of the CD4 + T-cells in the pancreas of AIP patients. Fig. S6. Flow cytometry gating strategy for Th and Tfh cells in co-culture experiments. Fig. S7. Heterogeneity of the CD8 + T-cells in the pancreas of AIP patients. Fig. S8. TCR analysis of all T cell subsets in the pancreas of AIP. Fig. S9. Comparison of the immune cell profile of AIP, CP and NI groups. Table S1. Clinical information of AIP patients and non-inflamed controls included for scRNA-seq. Table S2. Clinical information of AIP patients and non-inflamed controls included for flow cytometry validation. Table S3. Clinical information of AIP and CP patients and non-inflamed controls included for multiplex immunofluorescent. Table S4. Top10 marker genes for main cell types. Table S5. Top 10 marker genes for B cell subclusters. Table S6. Top 10 marker genes for macrophage subclusters. Table S7. Top 10 marker genes for CD4 + T cell subclusters. Table S8. Top 10 marker genes for CD8 + T cell subclusters. Table S9. Signature genes used to define functional gene sets.. [file 13073_2025_1567_MOESM1_ESM.pdf]

SUPPLEMENTARY FIGURES

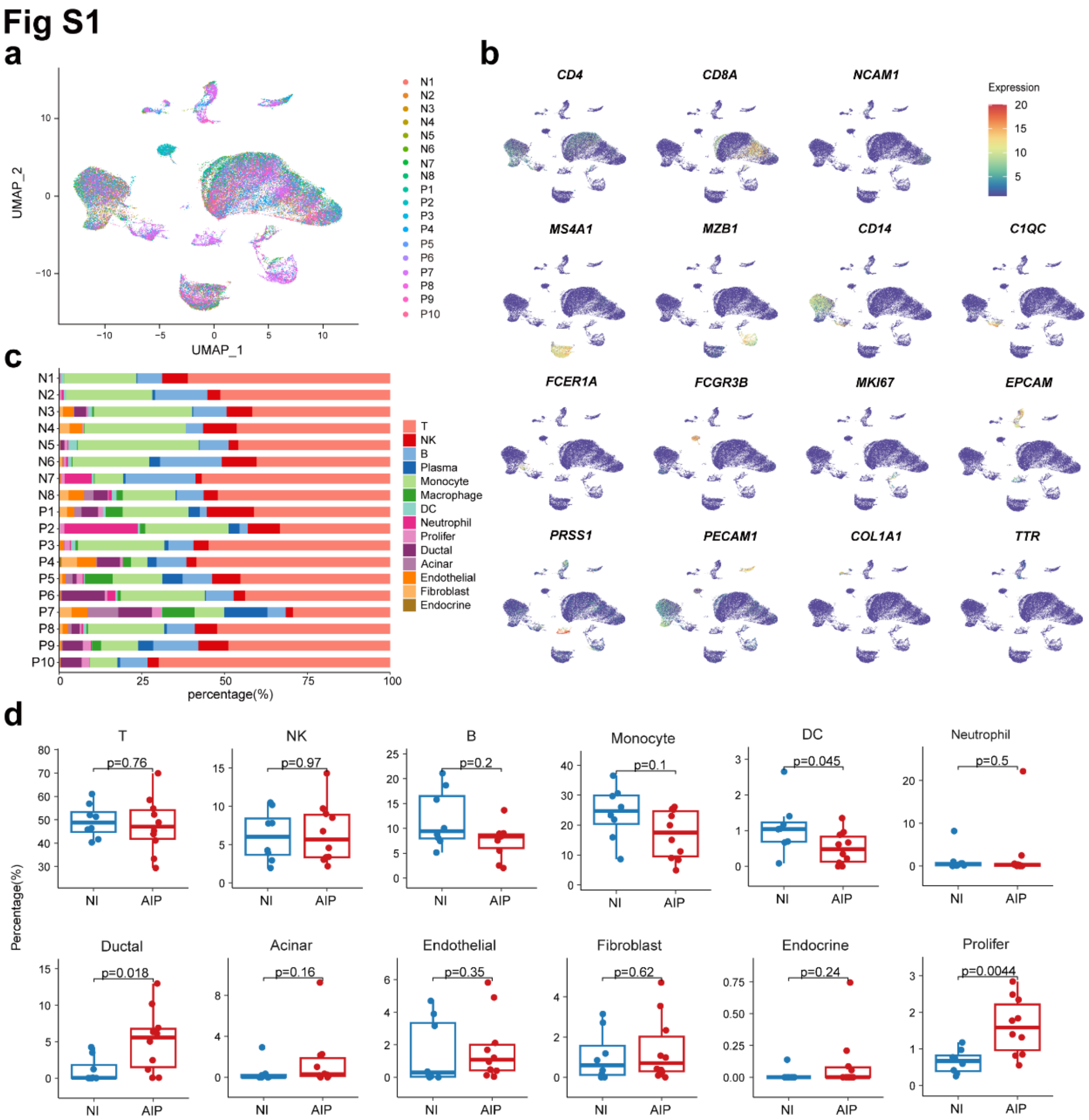

Fig S2

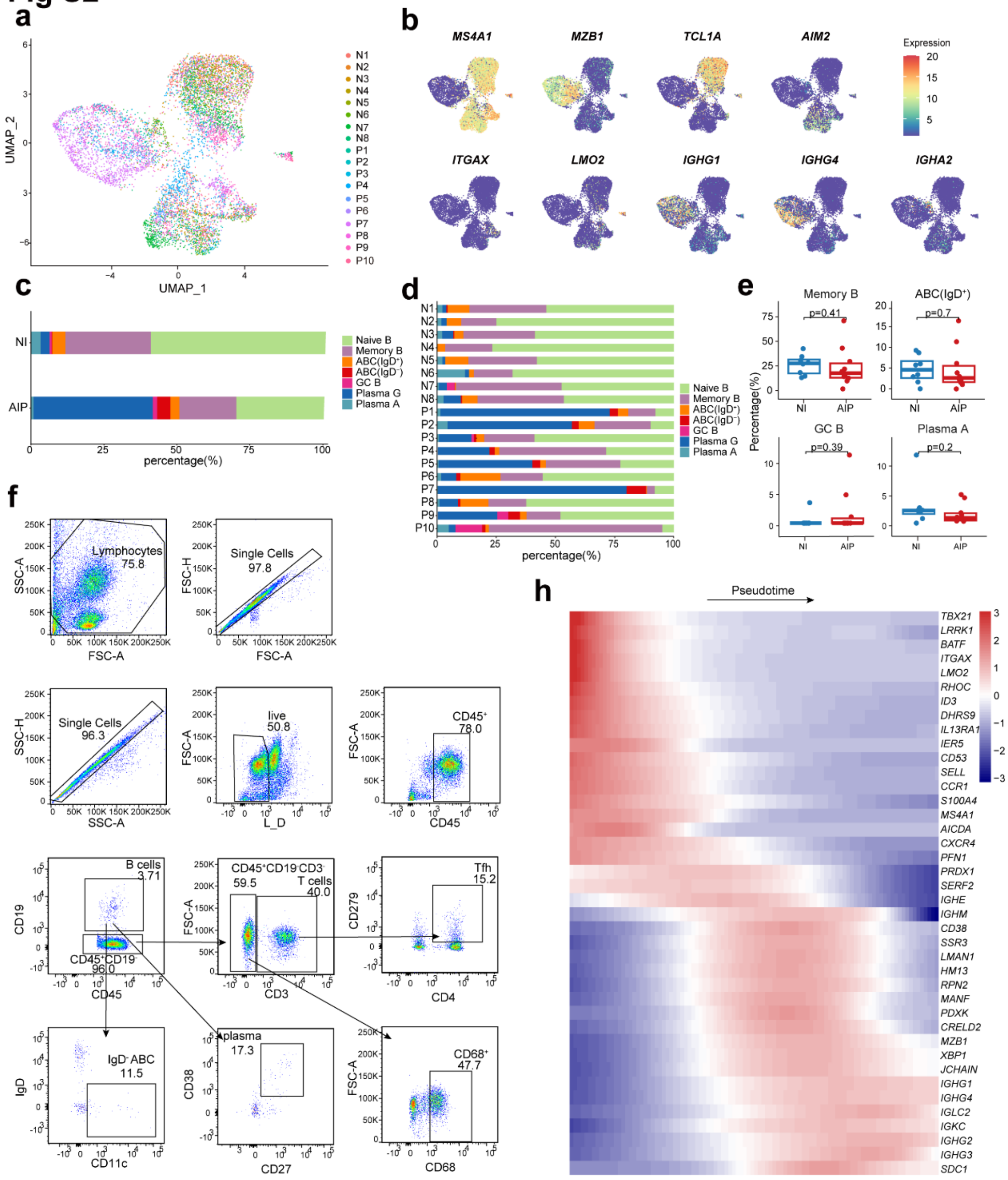
a
UMAP_1
UMAP_2
N1
N2
N3
N4
N5
N6
N7
N8
P1
P2
P3
P4
P5
P6
P7
P8
P9
P10
b
MS4A1
MZB1
TCL1A
AIM2
ITGAX
LMO2
IGHG1
IGHG4
IGHA2
Expression
c
NI
AIP
percentage(%)
Naive B
Memory B
ABC(IgD⁺)
ABC(IgD⁻)
GC B
Plasma G
Plasma A
d
percentage(%)
e
Memory B
ABC(IgD⁺)
GC B
Plasma A
Percentage(%)
p=0.41
p=0.7
p=0.39
p=0.2
f
Lymphocytes
75.8
Single Cells
97.8
Single Cells
96.3
live
50.8
CD45⁺
78.0
B cells
3.71
CD45⁺CD19⁻
96.0
CD45⁺CD19⁻CD3⁻
59.5
T cells
40.0
Tfh
15.2
IgD⁻ABC
11.5
plasma
17.3
CD68⁺
47.7
SSC-A
FSC-A
FSC-H
SSC-H
L_D
CD45
CD19
CD3
CD4
CD279
IgD
CD11c
CD38
CD27
CD68
h
Pseudotime
TBX21
LRRK1
BATF
ITGAX
LMO2
RHOC
ID3
DHRS9
IL13RA1
IER5
CD53
SELL
CCR1
S100A4
MS4A1
AICDA
CXCR4
PFN1
PRDX1
SERF2
IGHE
IGHM
CD38
SSR3
LMAN1
HM13
RPN2
MANF
PDXK
CRELD2
MZB1
XBP1
JCHAIN
IGHG1
IGHG4
IGLC2
IGKC
IGHG2
IGHG3
SDC1

Fig S3

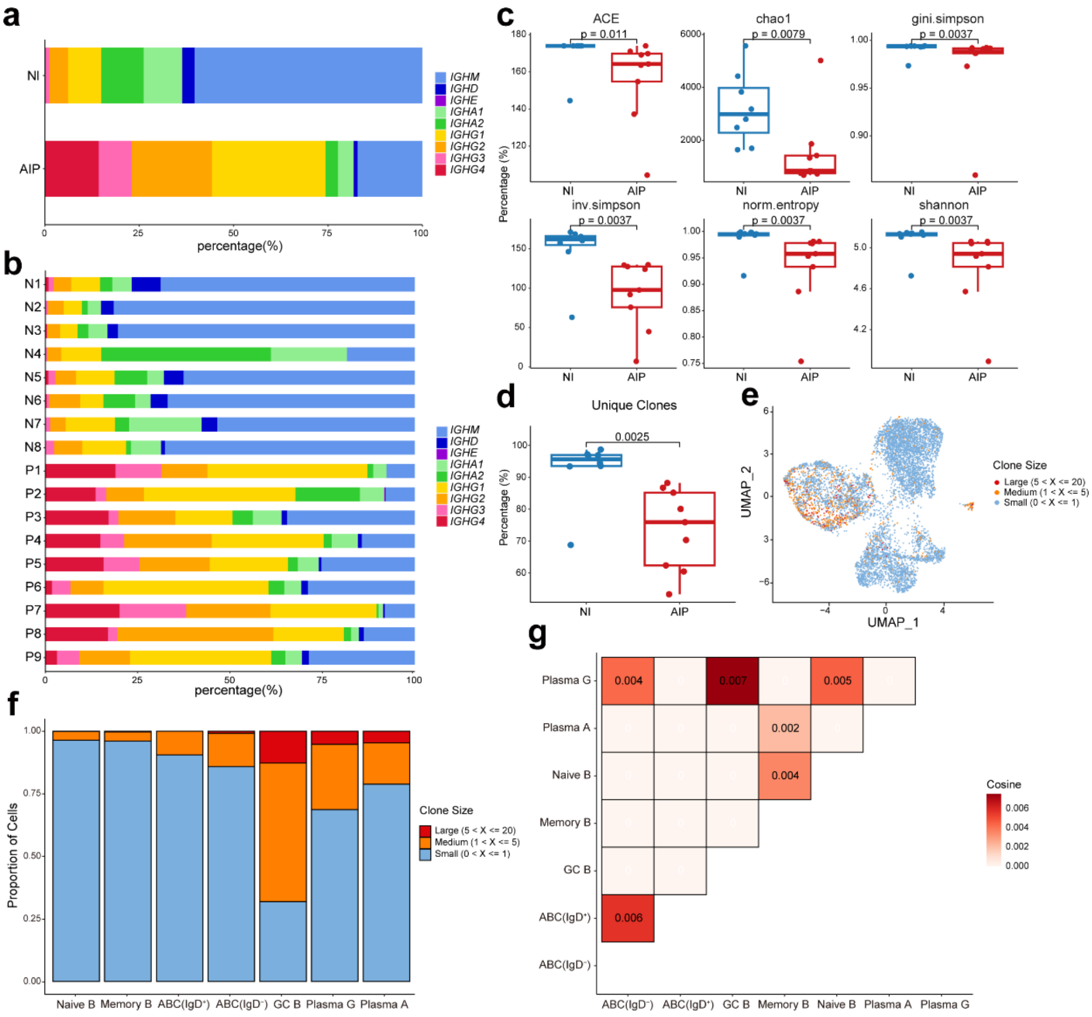
a
NI
AIP
percentage(%)
IGHM
IGHD
IGHE
IGHA1
IGHA2
IGHG1
IGHG2
IGHG3
IGHG4
b
N1
N2
N3
N4
N5
N6
N7
N8
P1
P2
P3
P4
P5
P6
P7
P8
P9
percentage(%)
c
ACE
p = 0.011
chao1
p = 0.0079
gini.simpson
p = 0.0037
inv.simpson
p = 0.0037
norm.entropy
p = 0.0037
shannon
p = 0.0037
Percentage (%)
d
Unique Clones
0.0025
e
UMAP_1
UMAP_2
Clone Size
Large (5 < X <= 20)
Medium (1 < X <= 5)
Small (0 < X <= 1)
f
Proportion of Cells
Naive B
Memory B
ABC(IgD⁺)
ABC(IgD⁻)
GC B
Plasma G
Plasma A
g
Cosine
Plasma G
Plasma A
Naive B
Memory B
GC B
ABC(IgD⁺)
ABC(IgD⁻)

Fig S4

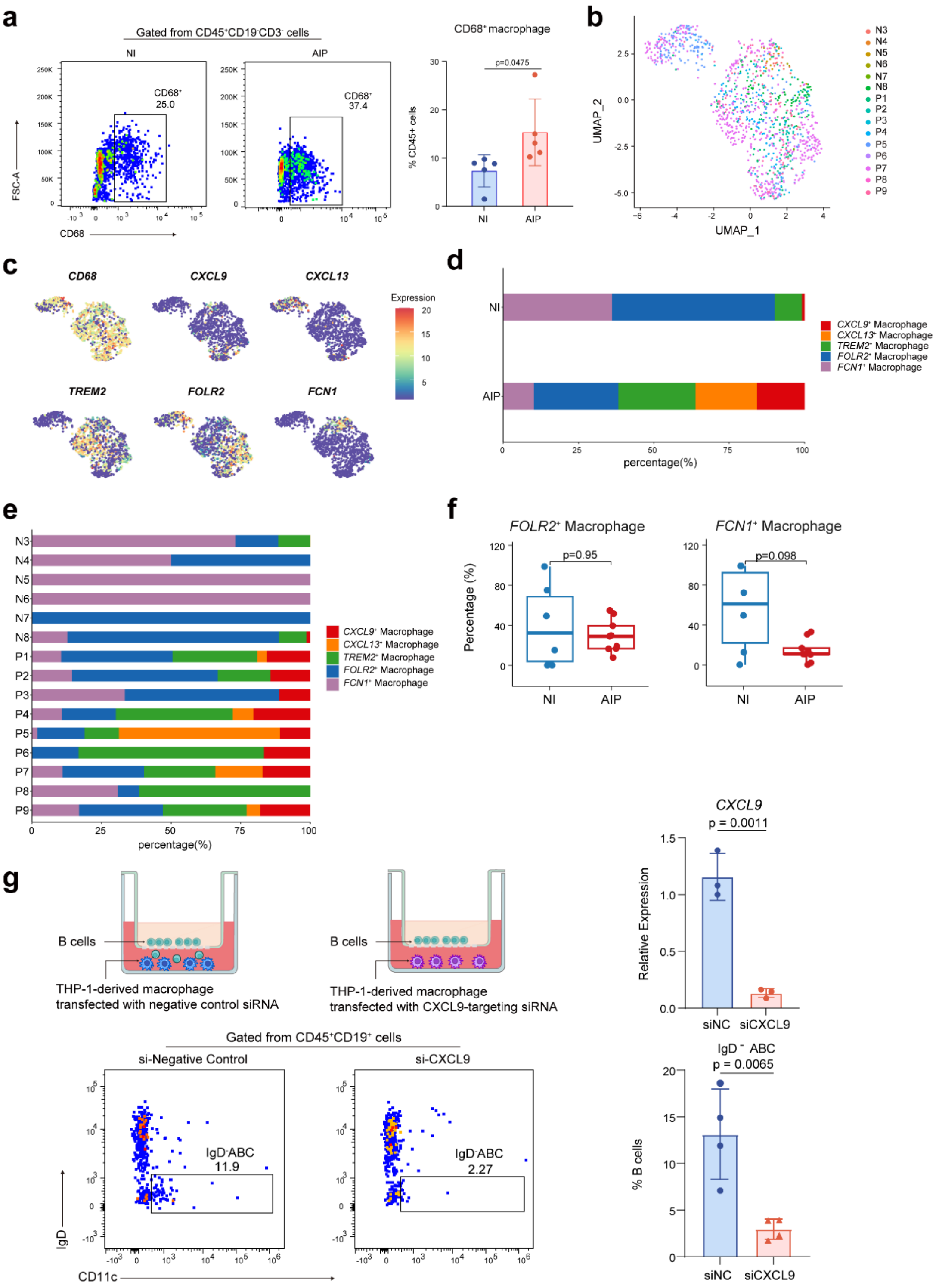

a
Gated from CD45⁺CD19⁻CD3⁻ cells
NI
AIP
CD68⁺ 25.0
CD68⁺ 37.4
FSC-A
CD68
CD68⁺ macrophage
p=0.0475
% CD45+ cells
b
UMAP_1
UMAP_2
N3
N4
N5
N6
N7
N8
P1
P2
P3
P4
P5
P6
P7
P8
P9
c
CD68
CXCL9
CXCL13
TREM2
FOLR2
FCN1
Expression
d
CXCL9⁺ Macrophage
CXCL13⁺ Macrophage
TREM2⁺ Macrophage
FOLR2⁺ Macrophage
FCN1⁺ Macrophage
percentage(%)
e
f
FOLR2⁺ Macrophage
FCN1⁺ Macrophage
p=0.95
p=0.098
Percentage (%)
g
B cells
THP-1-derived macrophage transfected with negative control siRNA
THP-1-derived macrophage transfected with CXCL9-targeting siRNA
CXCL9
p = 0.0011
Relative Expression
siNC
siCXCL9
Gated from CD45⁺CD19⁺ cells
si-Negative Control
si-CXCL9
IgD⁻ABC 11.9
IgD⁻ABC 2.27
IgD
CD11c
IgD⁻ ABC
p = 0.0065
% B cells

Fig S5

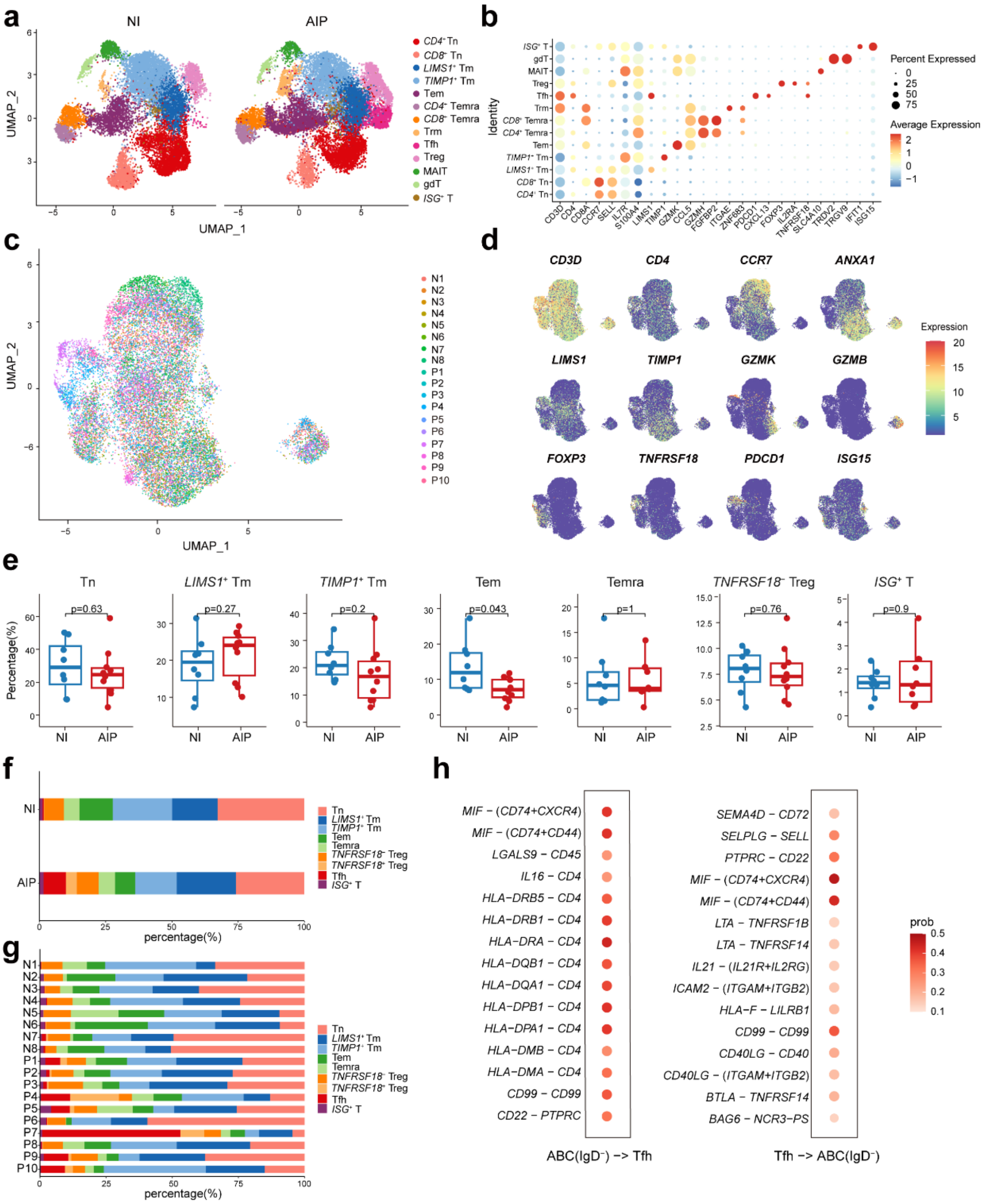
a
NI
AIP
UMAP_2
UMAP_1
b
Percent Expressed
Average Expression
Identity
c
d
Expression
e
Percentage(%)
f
percentage(%)
g
h
ABC(IgD⁻) -> Tfh
Tfh -> ABC(IgD⁻)
prob

Fig S6

a

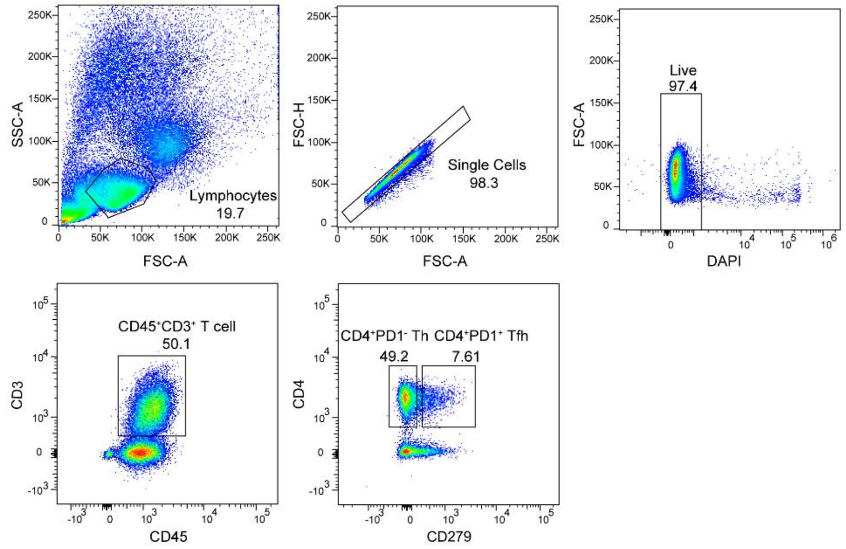
Lymphocytes
19.7
SSC-A
FSC-A
Single Cells
98.3
FSC-H
FSC-A
Live
97.4
FSC-A
DAPI
CD45+CD3+ T cell
50.1
CD3
CD45
CD4+PD1- Th
49.2
CD4+PD1+ Tfh
7.61
CD4
CD279

Fig S7

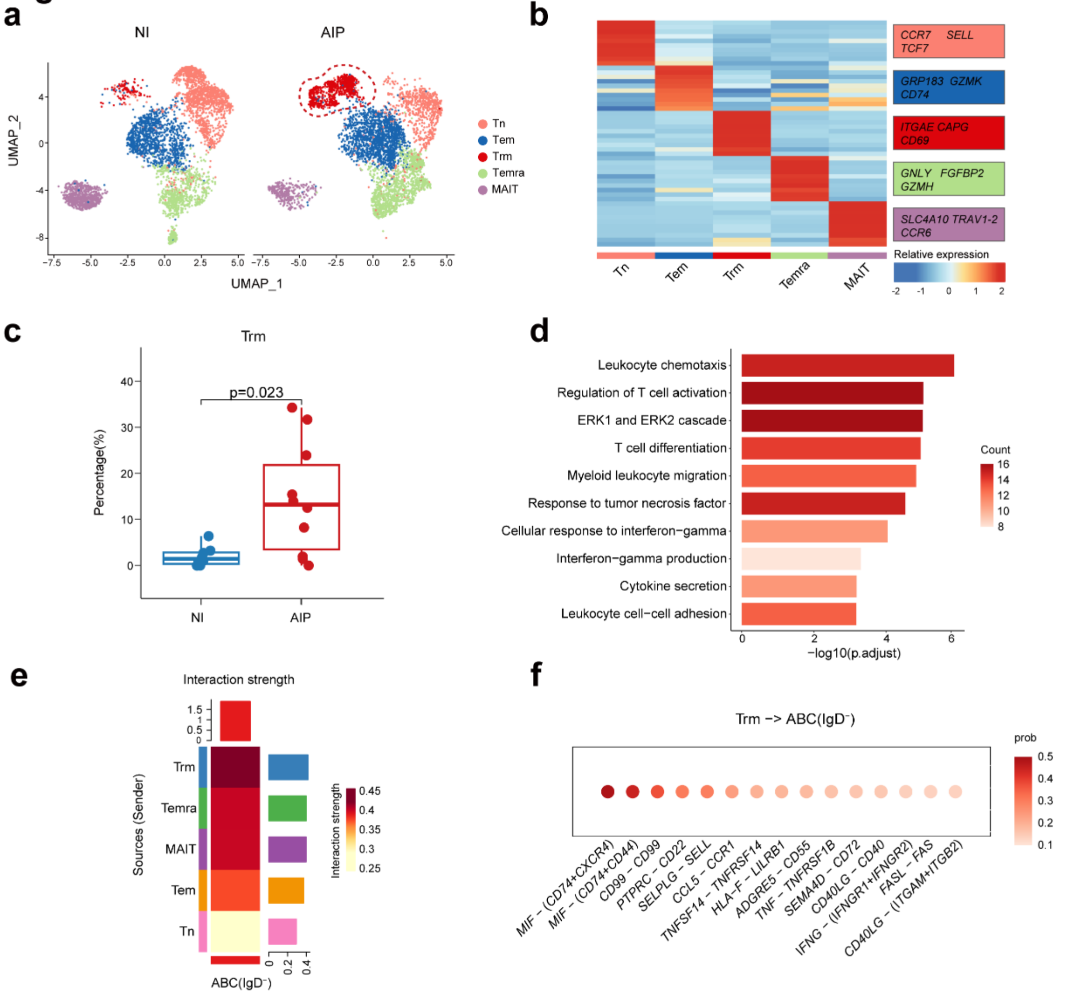
a
NI
AIP
UMAP_2
UMAP_1
Tn
Tem
Trm
Temra
MAIT
b
CCR7 SELL TCF7
GRP183 GZMK CD74
ITGAE CAPG CD69
GNLY FGFBP2 GZMH
SLC4A10 TRAV1-2 CCR6
Relative expression
Tn
Tem
Trm
Temra
MAIT
c
Trm
p=0.023
Percentage(%)
NI
AIP
d
Leukocyte chemotaxis
Regulation of T cell activation
ERK1 and ERK2 cascade
T cell differentiation
Myeloid leukocyte migration
Response to tumor necrosis factor
Cellular response to interferon−gamma
Interferon−gamma production
Cytokine secretion
Leukocyte cell−cell adhesion
−log10(p.adjust)
Count
e
Interaction strength
Sources (Sender)
Trm
Temra
MAIT
Tem
Tn
ABC(IgD⁻)
f
Trm −> ABC(IgD⁻)
prob
MIF − (CD74+CXCR4)
MIF − (CD74+CD44)
CD99 − CD99
PTPRC − CD22
SELPLG − SELL
CCL5 − CCR1
TNFSF14 − TNFRSF14
HLA−F − LILRB1
ADGRE5 − CD55
TNF − TNFRSF1B
SEMA4D − CD72
CD40LG − CD40
IFNG − (IFNGR1+IFNGR2)
FASL − FAS
CD40LG − (ITGAM+ITGB2)

Fig S8

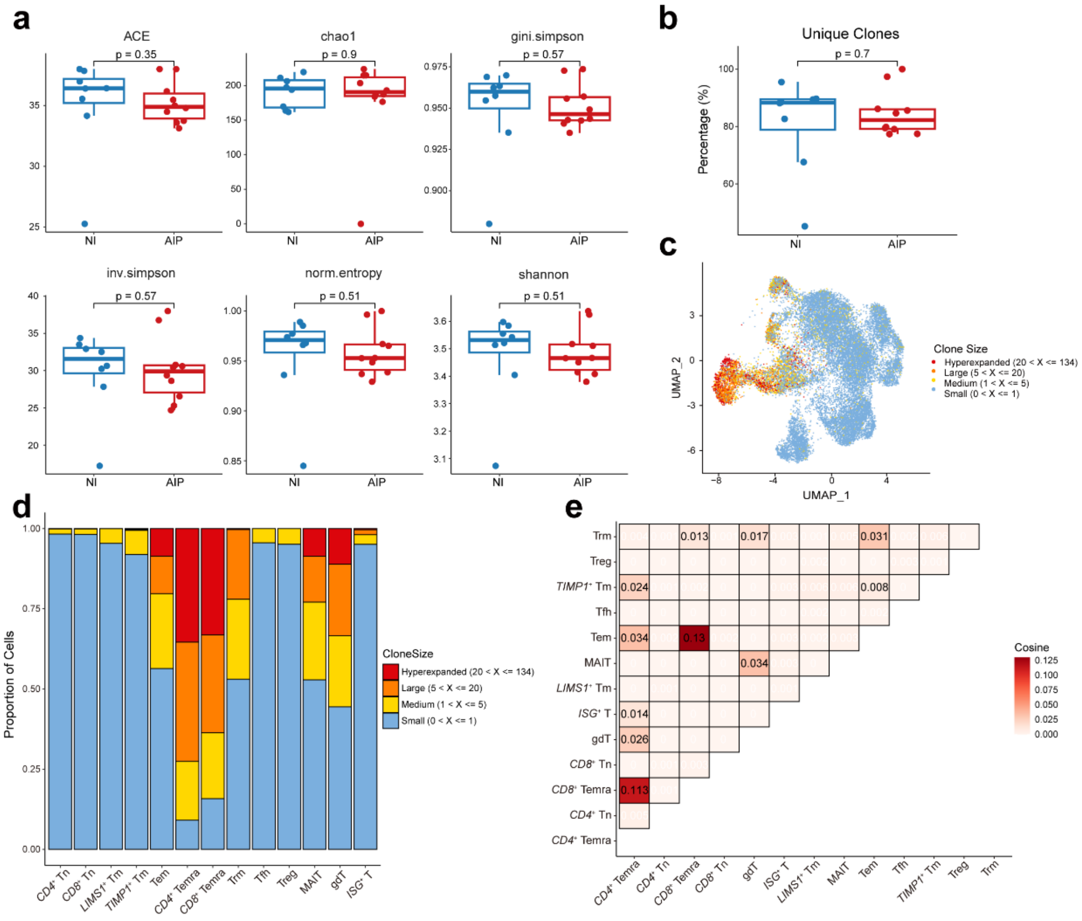
a
ACE
p = 0.35
chao1
p = 0.9
gini.simpson
p = 0.57
inv.simpson
p = 0.57
norm.entropy
p = 0.51
shannon
p = 0.51
NI
AIP
b
Unique Clones
p = 0.7
Percentage (%)
c
UMAP_1
UMAP_2
Clone Size
Hyperexpanded (20 < X <= 134)
Large (5 < X <= 20)
Medium (1 < X <= 5)
Small (0 < X <= 1)
d
Proportion of Cells
CloneSize
e
Cosine
Trm
Treg
TIMP1+ Tm
Tfh
Tem
MAIT
LIMS1+ Tm
ISG+ T
gdT
CD8+ Tn
CD8+ Temra
CD4+ Tn
CD4+ Temra

Fig S9

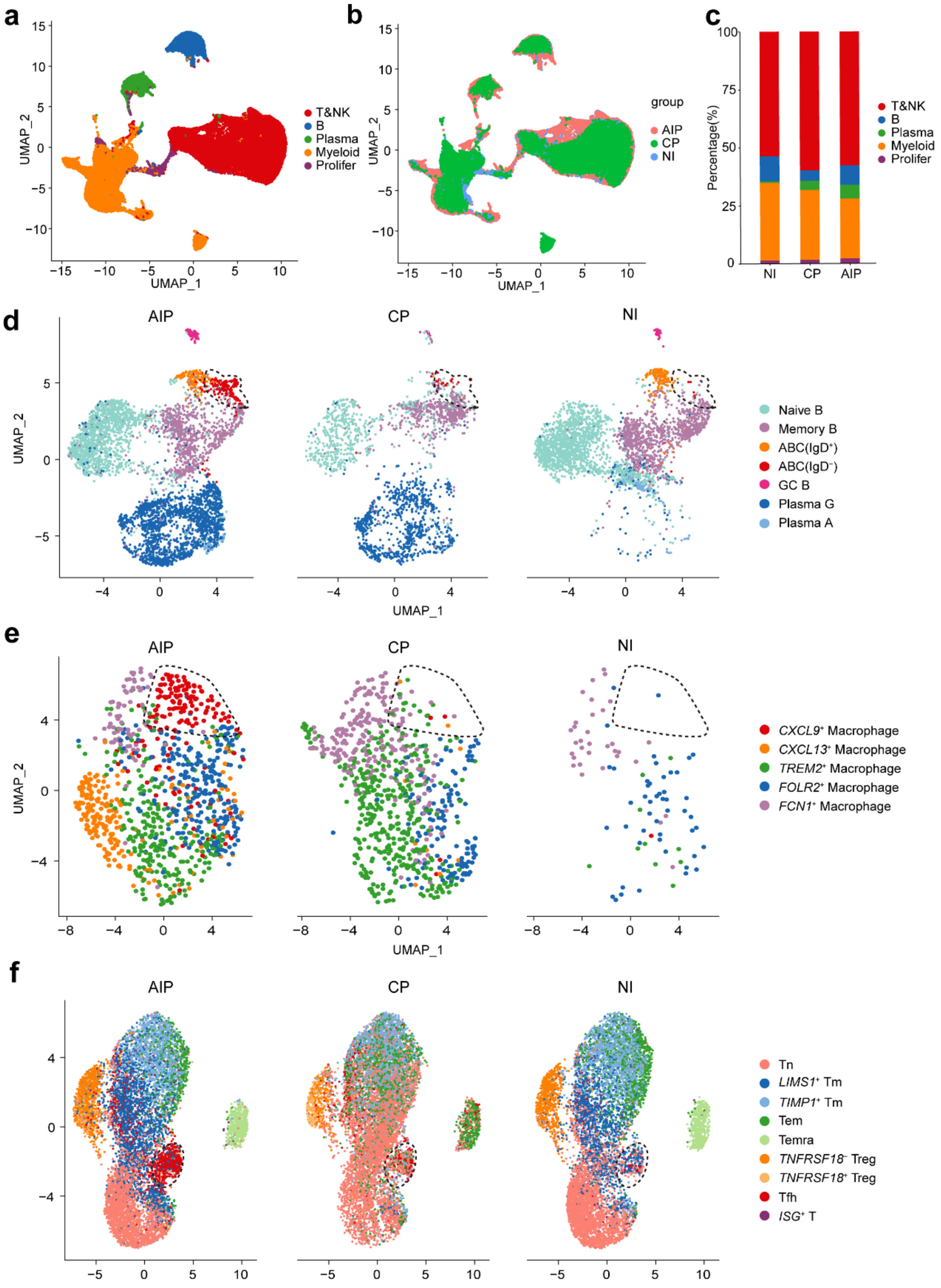
a
b
c
d
e
f
UMAP_1
UMAP_2
T&NK
B
Plasma
Myeloid
Prolifer
group
AIP
CP
NI
Percentage(%)
AIP
CP
NI
Naive B
Memory B
ABC(IgD⁺)
ABC(IgD⁻)
GC B
Plasma G
Plasma A
CXCL9⁺ Macrophage
CXCL13⁺ Macrophage
TREM2⁺ Macrophage
FOLR2⁺ Macrophage
FCN1⁺ Macrophage
Tn
LIMS1⁺ Tm
TIMP1⁺ Tm
Tem
Temra
TNFRSF18⁻ Treg
TNFRSF18⁺ Treg
Tfh
ISG⁺ T

SUPPLEMENTARY FIGURE LEGENDS

Fig S1. The landscape of AIP pancreatic microenvironment revealed by single-cell sequencing.

(a) UMAP plot showing all cells distribution of across 8 non-inflamed and 10 AIP pancreatic tissue samples.

(b) Feature plots showing the expression of canonical markers in major cell types.

(c) Bar plots showing percentage of major cell types across 18 samples.

(d) Comparison of frequencies of major cell types in non-inflamed pancreatic samples (n = 8) and AIP lesion pancreatic samples (n = 10). Statistical differences were determined by Wilcoxon rank-sum test.

Fig S2. Heterogeneity of the B cells in the pancreas of AIP patients.

(a) UMAP plot showing B cell subsets distribution across 8 non-inflamed and 10 AIP pancreatic tissue samples.

(b) Feature plots showing the expression of cluster-specific markers in B cell subsets.

(c) Bar graph showing percentage of B cell subsets in non-inflamed and AIP pancreatic tissues.

(d) Bar plots showing percentage of B cell subsets across 18 samples.

(e) Comparison of frequencies of B cell subsets in non-inflamed pancreatic samples (n = 8) and AIP lesion pancreatic samples (n = 10). Statistical differences were determined by Wilcoxon rank-sum test.

(f) Flow cytometry gating strategy for IgD^- ABCs, plasma cells, $CD68^+$ macrophages and Tfhs.

(g) Heatmap showing the dynamic expression (z score) changes of representative genes along the differentiation pseudotime of IgD^+ ABCs to IgD^- ABCs to plasma cells.

Fig S3. BCR analysis of all B cell subsets in the pancreas of AIP.

(a) Bar graph showing percentage of 9 immunoglobulin heavy chain isotypes of B cells in non-inflamed and AIP pancreatic tissues.

(b) Bar graph showing percentage of 9 immunoglobulin heavy chain isotypes of B cells across 17 samples.

(c) Boxplots comparing the clonal diversity of B cells in non-inflamed and AIP pancreatic tissues by ACE, chao1, gini.simpson, inverse simpson, norm entrophy and Shannon. Statistical difference was determined by Wilcoxon rank-sum test.

(d) Boxplots comparing the number of unique clones of B cells in non-inflamed and AIP pancreatic tissues. Statistical difference was determined by Wilcoxon rank-sum test.

(e) UMAP plot showing the clonesize of each B cell subset in AIP pancreatic tissue. Each dot represents a clonotype, and color represents the number of cells with this clone.

(f) Bar graph showing the clonesize of each B cell clone in AIP pancreatic tissue. Color represents the clone expansion degree.

(g) Heatmap showing the cloneoverlap among 7 B cell subsets with cosine similarity. Color represents cosine value.

Fig S4.Heterogeneity of the macrophages in the pancreas of AIP patients.

(a) Representative flow cytometry plots of $CD68^{+}$ macrophages in non-inflamed pancreas and AIP pancreatic lesions. Flow cytometry analysis revealed the proportion variation in $CD68^{+}$ to immune cells in non-inflamed pancreas and AIP pancreatic lesions. Points representing controls (n = 5) and AIP pancreatic samples (n = 5). Statistical differences were determined by two-tailed t-test.

(b) UMAP plot showing macrophage subsets distribution across non-inflamed and AIP pancreatic tissue samples (P10, N1, N2 contained none macrophages).

(c) Feature plots showing the expression of cluster-specific markers in macrophage subsets from AIP patients.

(d) Bar graph showing percentage of macrophage subsets in non-inflamed and AIP pancreatic tissues.

(e) Bar graph showing percentage of macrophage subsets across 15 samples.

(f) Comparison of frequencies of two macrophage subsets in non-inflamed pancreatic samples (n = 6) and AIP lesion pancreatic samples (n = 9). Statistical differences were determined by Wilcoxon rank-sum test.

(g) Schematic diagram and representative flow cytometry plots showing migrated IgD^- ABCs in transwell chemotaxis assays. THP-1-derived M1 macrophages were either subjected to CXCL9 silencing or negative control treatment (left panel). Validation of CXCL9 knockdown efficiency by qPCR (upper right, p = 0.0011). Flow quantification of migrated IgD^- ABCs demonstrates significantly reduced migration upon CXCL9 silencing compared to control (lower right, p = 0.0065). Data are presented as mean ± SD. Statistical differences were determined by two-tailed t-test.

Fig S5. Heterogeneity of the $CD4^+$ T-cells in the pancreas of AIP patients.

(a) UMAP plots showing all 13 T-cell subsets in non-inflamed and AIP lesion pancreatic tissues.

(b) Dot plots of representative markers in the indicated T-cell subsets. The average gene expression and percentage of cells expressed are shown by dot color and size, respectively.

(c) UMAP plot showing $CD4^+$ T-cell subsets distribution across 8 non-inflamed and 10 AIP pancreatic tissue samples.

(d) Feature plots showing the expression of cluster-specific markers in $CD4^+$ T-cell subsets.

(e) Comparison of frequencies of $CD4^+$ T-cell subsets in non-inflamed pancreatic samples (n = 8) and AIP lesion pancreatic samples (n = 10). Statistical differences were determined by Wilcoxon rank-sum test.

(f) Bar graph showing percentage of $CD4^+$ T-cell subsets in non-inflamed and AIP

pancreatic tissues.

(g) Bar graph showing percentage of $CD4^+$ T-cell subsets across 18 samples.

(h) Dot plot showing the top 15 receptor–ligand pairs between IgD^- ABCs and Tfhs. Colour represents the probability of interaction.

Fig S6. Flow cytometry gating strategy for Th and Tfh cells in co-culture experiments.

(a) Flow cytometry gating strategy for Th ($CD45^+CD3^+CD4^+PD1^-$) and Tfh cells ($CD45^+CD3^+CD4^+PD1^+$) in co-culture experiments.

Fig S7. Heterogeneity of the $CD8^+$ T-cells in the pancreas of AIP patients.

(a) UMAP plots showing 5 $CD8^+$ T-cell subsets in non-inflamed and AIP lesion pancreatic tissues in different colour. Trm with significant quantitative changes are circled by dotted lines.

(b) Heatmap showing the relative expression (z score) of marker genes in subclustered $CD8^+$ T-cell subsets. Clusters are colored as in (a). Three representative genes of each cluster were listed in the right rectangles and all top ten maker genes were listed in Supplementary Table 10.

(c) Box graph comparing the percentage of Trm in non-inflamed and AIP pancreatic tissues. Statistical differences were determined by Wilcoxon rank-sum test.

(d) Representative enriched GO biological process terms of DEGs expressed in Trm. A hypergeometric test was performed with FDR adjusted P values.

(e) Heatmap showing the differential interaction strength among all $CD8^+$ T-cell subsets and IgD^- ABCs.

(f) Dot plot showing the top 15 receptor–ligand pairs from Trm to IgD^- ABCs. Colour represents the probability of interaction.

Fig S8. TCR analysis of all T cell subsets in the pancreas of AIP.

(a) Boxplots comparing the clonal diversity of T-cells in non-inflamed and AIP pancreatic tissues by ACE, chao1, gini.simpson, inverse simpson, norm.entrophy

and Shannon. Statistical difference was determined by Wilcoxon rank-sum test.

(b) Boxplots comparing the number of unique clones of T-cells in non-inflamed and AIP pancreatic tissues. Statistical difference was determined by Wilcoxon rank-sum test.

(c) UMAP plot showing the clonesize of each T-cell subset in AIP pancreatic tissues. Each dot represents a clonotype, and color represents the number of cells with this clone. The UMAP plot embeddings are same to the Supplementary Fig 5a.

(d) Bar graph showing the clonesize of each T-cell clone in AIP pancreatic tissues. Color represents the clone expansion degree.

(e) Heatmap showing the cloneoverlap among 13 T-cell subsets with cosine similarity. Color represents cosine value.

Fig S9. Comparison of the immune cell profile of AIP, CP and non-inflamed control groups.

(a) UMAP plots showing immune cell types from single cell RNA sequencing dataset of CP (GSE165045) with the dataset from AIP (this work).

(b) UMAP plots showing all cells distribution across 3 groups: AIP, CP and non-inflamed.

(c) Bar plots showing percentage of immune cell types across 3 groups.

(d) UMAP plots showing B cell subsets in non-inflamed, CP and AIP lesion pancreatic tissues in different colour. IgD$^-$ABCs are circled by dotted lines with significant quantitative changes.

(e) UMAP plots showing macrophages cell subsets in non-inflamed, CP and AIP lesion pancreatic tissues in different colour. *CXCL9*$^+$ macrophages are circled by dotted lines with significant quantitative changes.

(f) UMAP plots showing CD4$^+$ T-cell subsets in non-inflamed, CP and AIP lesion pancreatic tissues in different colour. Tfhs are circled by dotted lines with significant quantitative changes.

SUPPLEMENTARY TABLES:

Table S1. Clinical information of AIP patients and non-inflamed controls included for scRNA-seq (cohort1).

<table>
<tr><th>Group</th><th colspan="3">AIP (n = 10)</th><th>NI (n = 8)</th><th>P value</th></tr>
<tr><td>Age (y/o, mean ± SD)</td><td colspan="3">66.7 ± 8.8</td><td>65.8 ± 10.7</td><td>0.84</td></tr>
<tr><td>Gender (male/female)</td><td colspan="3">10/0</td><td>3/5</td><td>0.0065</td></tr>
<tr><td>Body mass index (kg/m2, mean ± SD)</td><td colspan="3">21.0 ± 2.5</td><td>21.5 ± 1.7</td><td>0.66</td></tr>
<tr><td>IgG4 (g/L, mean ± SD)</td><td colspan="3">7.9 ± 6.2</td><td>0.8 ± 0.3</td><td>0.0057</td></tr>
<tr><td>TBIL (μmol/L, mean ± SD)</td><td colspan="3">57.5 ± 66.8</td><td>13.5 ± 2.7</td><td>0.080</td></tr>
<tr><td>HbAlc (%, mean ± SD)</td><td colspan="3">6.1 ± 0.5</td><td>7.2 ± 1.8</td><td>0.087</td></tr>
<tr><td>Glucose (mmol/L, mean ± SD)</td><td colspan="3">5.9 ± 1.2</td><td>7.6 ± 4.4</td><td>0.24</td></tr>
<tr><td>MRCP use (Yes/No)</td><td colspan="3">10 / 0</td><td>8 / 0</td><td>>0.9999</td></tr>
<tr><td>Pancreatic duct strictures/ Narrowing (Yes/No)</td><td colspan="3">10 / 0</td><td>0 / 8</td><td><0.0001</td></tr>
<tr><td>Dilated pancreatic duct (Yes/No)</td><td colspan="3">0 / 10</td><td>3 / 5</td><td>0.0065</td></tr>
<tr><td rowspan="2">Other organ involvement</td><td>salivary glands</td><td>bile duct</td><td>none</td><td>none</td><td></td></tr>
<tr><td>1</td><td>7</td><td>2</td><td>8</td><td></td></tr>
</table>

AIP, autoimmune pancreatitis; NI, non-inflamed control; y/o, years old; TBIL, total bilirubin; MRCP, magnetic resonance cholangiopancreatography.

Table S2. Clinical information of AIP patients and non-inflamed controls included for flow cytometry validation (cohort2).

Group	AIP (n = 5)	NI (n = 5)	P value
Age (y/o, mean ± SD)	68.4 ± 4.5	68.8 ± 4.4	0.89
Gender (male/female)	5 / 0	2 / 3	0.17

**Body mass index (kg/m2, mean ± SD)**	20.7 ± 1.1			21.8 ± 2.9	0.48
**IgG4 (g/L, mean ± SD)**	13.2 ± 3.5			0.7 ± 0.3	<0.0001
**TBIL (μmol/L, mean ± SD)**	70.8 ± 92.5			31.9 ± 18.4	0.38
**HbAlc (%, mean ± SD)**	6.5 ± 1.0			6.7 ± 1.3	0.81
**Glucose (mmol/L, mean ± SD)**	5.9 ± 0.7			7.3 ± 4.5	0.49
**MRCP use (Yes/No)**	5 / 0			3 / 2	0.44
**Pancreatic duct strictures/ Narrowing (Yes/No)**	4 / 1			0 / 5	0.048
**Dilated pancreatic duct (Yes/No)**	1 / 4			3 / 2	0.52
**Other organ involvement**	salivary glands	bile duct	none	none	
	1	3	1	5	

AIP, autoimmune pancreatitis; NI, non-inflamed control; y/o, years old; TBIL, total bilirubin; MRCP, magnetic resonance cholangiopancreatography.

Table S3. Clinical information of AIP and CP patients and non-inflamed controls included for multiplex immunofluorescent (cohort3).

**Group**	**AIP (n = 5)**	**NI (n = 5)**	**CP (n = 10)**	**P value**
**Age (y/o, mean ± SD)**	68.4 ± 9.2	59.2 ± 11.8	55.5 ± 13.4	0.18
**Gender (male/female)**	5 / 0	4 / 1	8 / 2	0.78
**Body mass index (kg/m2, mean ± SD)**	23.7 ± 4.1	24.5 ± 2.6	22.2 ± 4.2	0.51
**IgG4 (g/L, mean ± SD)**	12.0 ± 8.2	0.9 ± 0.3	0.4 ± 0.4	0.0002
**TBIL (μmol/L, mean ± SD)**	40.0 ± 31.0	26.3 ± 6.9	13.1 ± 5.0	0.020
**HbAlc (%, mean ± SD)**	6.8 ± 1.8	5.8 ± 1.4	5.8 ± 0.7	0.32
**Glucose (mmol/L, mean ± SD)**	6.7 ± 2.2	4.7 ± 1.4	5.9 ± 1.0	0.12
**MRCP use (Yes/No)**	5 / 0	2 / 3	10 / 0	0.018

**Pancreatic duct strictures/ Narrowing (Yes/No)**	5 / 0			0 / 5	3 / 7	0.042
**Dilated pancreatic duct (Yes/No)**	1 / 4			0 / 5	3 / 7	0.046
**Other organ involvement**	salivary glands	bile duct	none	none	none	
	1	3	1	5	10	

AIP, autoimmune pancreatitis; NI, non-inflamed control; y/o, years old; TBIL, total bilirubin; MRCP, magnetic resonance cholangiopancreatography.

Table S4. Top10 marker genes for main cell types.

avg_log2FC	pct.1	pct.2	p_val_adj	cluster	gene
3.328253	0.693	0.019	0	T	*IL7R*
2.850105	0.901	0.074	0	T	*CD3E*
2.716721	0.833	0.041	0	T	*CD3D*
2.454234	0.921	0.191	0	T	*IL32*
2.387949	0.254	0.017	0	T	*GZMK*
2.081902	0.541	0.025	0	T	*TCF7*
2.024283	0.644	0.032	0	T	*CD3G*
1.941522	0.654	0.084	0	T	*CD2*
1.900888	0.68	0.102	0	T	*LINC00861*
1.805408	0.788	0.374	0	T	*LDHB*
4.614775	0.964	0.064	0	NK	*GNLY*
4.010781	0.972	0.065	0	NK	*GZMB*
3.701262	0.985	0.16	0	NK	*PRF1*
3.659085	1	0.196	0	NK	*NKG7*
3.534797	0.896	0.05	0	NK	*FGFBP2*
3.03929	0.963	0.171	0	NK	*CTSW*
2.963612	0.727	0.045	0	NK	*SPON2*
2.935442	0.988	0.215	0	NK	*CST7*
2.920546	0.906	0.056	0	NK	*KLRD1*
2.822639	0.886	0.091	0	NK	*GZMH*
4.304013	0.925	0.018	0	B	*MS4A1*
4.149267	0.926	0.039	0	B	*CD79A*
3.213096	0.533	0.002	0	B	*TCL1A*
2.894631	0.66	0.008	0	B	*IGHM*
2.829539	0.996	0.819	0	B	*CD74*
2.715242	0.729	0.008	0	B	*BANK1*
2.695605	0.987	0.396	0	B	*HLA-DRA*
2.634954	0.929	0.223	0	B	*HLA-DQA1*
2.569427	0.75	0.071	0	B	*CD79B*

2.553981	0.706	0.048	0	B	*RALGPS2*
8.220063	0.358	0.045	0	Plasma	*IGHV5-51*
7.618999	0.246	0.019	0	Plasma	*IGHV1-69D*
7.40456	0.188	0.022	0	Plasma	*IGKV3-15*
7.189999	0.226	0.038	0	Plasma	*IGKV3-20*
6.946428	0.157	0.02	0	Plasma	*IGKV1-39*
6.911855	0.184	0.025	0	Plasma	*IGKV3-11*
6.758747	0.163	0.02	0	Plasma	*IGKV1-5*
6.754394	0.119	0.01	0	Plasma	*IGKV1-12*
6.297906	0.116	0.015	0	Plasma	*IGHV4-39*
6.525502	0.112	0.036	3.90E-90	Plasma	*IGLV2-14*
4.478787	0.974	0.068	0	Monocyte	*LYZ*
3.855829	0.932	0.016	0	Monocyte	*FCN1*
3.655291	0.977	0.075	0	Monocyte	*S100A9*
3.417869	0.965	0.19	0	Monocyte	*IFI30*
3.366821	0.752	0.016	0	Monocyte	*VCAN*
3.257326	0.939	0.058	0	Monocyte	*S100A8*
3.200226	0.961	0.126	0	Monocyte	*AIF1*
3.03055	0.966	0.283	0	Monocyte	*CTSS*
3.007333	0.938	0.095	0	Monocyte	*LST1*
2.988649	0.992	0.179	0	Monocyte	*TYROBP*
5.988474	0.794	0.027	0	Macrophage	*APOE*
5.4047	0.78	0.013	0	Macrophage	*C1QC*
5.34216	0.766	0.015	0	Macrophage	*C1QB*
5.26871	0.752	0.022	0	Macrophage	*APOC1*
5.074369	0.76	0.021	0	Macrophage	*C1QA*
3.88194	0.299	0.043	0	Macrophage	*SPP1*
3.160852	0.446	0.042	0	Macrophage	*LGMN*
3.157601	0.37	0.059	0	Macrophage	*SELENOP*
2.996022	0.995	0.966	0	Macrophage	*FTL*
2.976082	0.655	0.305	0	Macrophage	*CTSB*
3.229171	1	0.328	0	DC	*CST3*
3.097933	0.837	0.003	0	DC	*FCER1A*
2.859592	0.995	0.282	0	DC	*HLA-DQA1*
2.854756	0.998	0.315	0	DC	*HLA-DQB1*
2.828623	1	0.446	0	DC	*HLA-DRA*
2.803291	1	0.514	0	DC	*HLA-DRB1*
2.757441	1	0.529	0	DC	*HLA-DPA1*
2.741929	1	0.552	0	DC	*HLA-DPB1*
2.586652	0.8	0.018	0	DC	*CLEC10A*
2.569186	0.81	0.022	0	DC	*CD1C*
5.376563	0.723	0.028	0	Neutrophil	*G0S2*
5.081403	0.89	0.018	0	Neutrophil	*FCGR3B*
4.812581	0.955	0.149	0	Neutrophil	*CSF3R*

4.754292	0.995	0.778	0	Neutrophil	*IFITM2*
4.502829	0.972	0.517	0	Neutrophil	*SAT1*
4.414952	0.8	0.123	0	Neutrophil	*SLC25A37*
4.263855	0.851	0.154	0	Neutrophil	*NAMPT*
4.16724	0.637	0.008	0	Neutrophil	*CMTM2*
4.087325	0.996	0.206	0	Neutrophil	*S100A8*
4.046193	0.614	0.02	0	Neutrophil	*TNFRSF10C*
3.719273	0.896	0.125	0	Prolifer	*STMN1*
3.261524	0.964	0.485	0	Prolifer	*TUBA1B*
2.843311	0.465	0.016	0	Prolifer	*HIST1H1B*
2.818592	0.856	0.302	0	Prolifer	*HMGB2*
2.804206	0.911	0.461	0	Prolifer	*TUBB*
2.718871	0.699	0.008	0	Prolifer	*TYMS*
2.637625	0.626	0.006	0	Prolifer	*RRM2*
2.456697	0.929	0.552	0	Prolifer	*HMGN2*
2.43083	0.919	0.493	0	Prolifer	*H2AFZ*
2.960779	0.837	0.558	1.86E-192	Prolifer	*HIST1H4C*
5.699415	0.894	0.029	0	Ductal	*FXYD2*
5.122588	0.532	0.006	0	Ductal	*LCN2*
4.852893	0.947	0.143	0	Ductal	*ANXA4*
4.74078	0.858	0.052	0	Ductal	*CLU*
4.393159	0.282	0.008	0	Ductal	*TFF3*
4.16213	0.736	0.023	0	Ductal	*SPP1*
4.114722	0.768	0.005	0	Ductal	*MMP7*
4.062322	0.824	0.011	0	Ductal	*CLDN10*
4.053753	0.663	0.018	0	Ductal	*SERPINA3*
3.900249	0.783	0.009	0	Ductal	*DEFB1*
8.053028	0.996	0.128	0	Acinar	*PRSS1*
7.918299	0.992	0.091	0	Acinar	*PRSS2*
7.821633	0.992	0.067	0	Acinar	*CTRB1*
7.766772	0.959	0.101	0	Acinar	*CLPS*
7.484415	0.993	0.078	0	Acinar	*CTRB2*
7.48027	0.99	0.074	0	Acinar	*CELA3A*
7.337335	0.846	0.045	0	Acinar	*REG3A*
7.191035	0.983	0.062	0	Acinar	*CELA3B*
7.145869	0.972	0.051	0	Acinar	*PLA2G1B*
7.040419	0.973	0.066	0	Acinar	*PNLIP*
5.548808	0.916	0.102	0	Endothelial	*FABP5*
5.494054	0.927	0.002	0	Endothelial	*PLVAP*
5.024204	0.945	0.014	0	Endothelial	*SPARCL1*
4.794008	0.969	0.026	0	Endothelial	*IFI27*
4.663465	0.872	0.112	0	Endothelial	*CD320*
4.559707	0.929	0.003	0	Endothelial	*RAMP2*
4.540842	0.943	0.027	0	Endothelial	*A2M*

4.333997	0.939	0.046	0	Endothelial	*IGFBP4*
4.222944	0.942	0.108	0	Endothelial	*IGFBP7*
4.117533	0.843	0.005	0	Endothelial	*CLEC3B*
6.340213	0.506	0.005	0	Fibroblast	*APOD*
5.723071	0.864	0.024	0	Fibroblast	*MGP*
5.519449	0.733	0.002	0	Fibroblast	*DCN*
5.436603	0.921	0.114	0	Fibroblast	*IGFBP7*
4.921818	0.543	0.002	0	Fibroblast	*LUM*
4.886372	0.546	0.002	0	Fibroblast	*C7*
4.688834	0.749	0.002	0	Fibroblast	*COL3A1*
4.687742	0.394	0.001	0	Fibroblast	*SFRP2*
4.622257	0.537	0.019	0	Fibroblast	*TAGLN*
4.578125	0.893	0.331	0	Fibroblast	*TIMP1*
11.01838	0.551	0.015	0	Endocrine	*INS*
8.310139	0.128	0	0	Endocrine	*SST*
7.70486	0.872	0.009	0	Endocrine	*TTR*
6.03737	0.872	0	0	Endocrine	*CHGA*
5.764129	0.974	0.026	0	Endocrine	*PCSK1N*
5.033084	0.474	0.008	0	Endocrine	*RBP4*
4.770029	0.115	0	0	Endocrine	*GCG*
4.112829	0.821	0.003	0	Endocrine	*SCG5*
3.869212	0.808	0.002	0	Endocrine	*CHGB*
6.47524	0.154	0.004	2.43E-82	Endocrine	*GHRL*

Table S5. Top 10 marker genes for B cell subclusters.

avg_log2FC	pct.1	pct.2	p_val_adj	cluster	gene
3.148475	0.867	0.058	0	Naive B	*TCL1A*
2.052369	0.73	0.149	0	Naive B	*FCER2*
1.762946	0.491	0.065	0	Naive B	*IL4R*
1.500608	0.783	0.313	0	Naive B	*HVCN1*
1.388519	0.469	0.102	0	Naive B	*BACH2*
1.363144	0.952	0.601	0	Naive B	*BTG1*
1.343637	0.471	0.069	0	Naive B	*IGHD*
1.254136	0.811	0.362	0	Naive B	*SELL*
1.253898	0.598	0.211	0	Naive B	*NIBAN3*
1.324218	0.529	0.203	1.74E-235	Naive B	*CD69*
1.295442	0.452	0.065	0	Memory B	*AIM2*
1.382318	0.332	0.049	2.71E-274	Memory B	*LINC01781*
1.064817	0.435	0.124	9.47E-216	Memory B	*MARCKS*
0.892274	0.331	0.07	2.24E-208	Memory B	*TNFRSF13B*
1.32978	0.674	0.34	8.39E-198	Memory B	*CRIP1*
0.983839	0.94	0.783	1.57E-192	Memory B	*RPS29*
0.930023	0.512	0.19	7.19E-182	Memory B	*CD82*
1.161924	0.319	0.106	9.22E-130	Memory B	*ITGB1*

1.033656	0.397	0.187	7.38E-96	Memory B	*S100A4*
0.996451	0.38	0.17	8.39E-95	Memory B	*AHNAK*
2.055303	0.766	0.117	1.57E-253	ABC(IgD$^+$)	*FGR*
1.517679	0.601	0.076	9.54E-218	ABC(IgD$^+$)	*HCK*
1.700789	0.759	0.142	3.72E-197	ABC(IgD$^+$)	*ZEB2*
1.744609	0.722	0.167	1.60E-143	ABC(IgD$^+$)	*FCRL3*
2.091403	0.858	0.356	5.57E-127	ABC(IgD$^+$)	*CIB1*
1.583645	0.718	0.199	6.88E-124	ABC(IgD$^+$)	*DAPP1*
1.537539	0.513	0.099	2.35E-117	ABC(IgD$^+$)	*PPP1R14A*
1.877888	0.934	0.399	3.11E-115	ABC(IgD$^+$)	*CRIP1*
1.694069	0.905	0.546	5.42E-89	ABC(IgD$^+$)	*IFI30*
1.621046	0.633	0.219	1.13E-80	ABC(IgD$^+$)	*FCRL5*
1.958951	0.563	0.025	0	ABC(IgD$^-$)	*DHRS9*
2.178692	0.762	0.222	1.26E-99	ABC(IgD$^-$)	*S100A4*
1.582323	0.628	0.153	5.02E-87	ABC(IgD$^-$)	*ANXA4*
1.801667	0.541	0.132	9.75E-77	ABC(IgD$^-$)	*HCST*
1.892367	0.957	0.74	1.54E-70	ABC(IgD$^-$)	*GAPDH*
1.739665	0.727	0.265	1.25E-68	ABC(IgD$^-$)	*CAPG*
1.68604	0.649	0.242	1.15E-55	ABC(IgD$^-$)	*S100A11*
1.686474	0.965	0.745	1.57E-54	ABC(IgD$^-$)	*ACTG1*
1.740443	0.81	0.552	2.29E-43	ABC(IgD$^-$)	*IFI30*
1.587897	0.294	0.061	2.12E-41	ABC(IgD$^-$)	*IGHE*
3.203751	0.752	0.025	0	GC B	*RGS13*
3.274614	0.812	0.053	2.09E-236	GC B	*MEF2B*
2.827075	0.743	0.073	6.53E-145	GC B	*LMO2*
2.530175	0.624	0.054	4.46E-129	GC B	*ELL3*
2.512299	0.653	0.099	4.58E-82	GC B	*HMCES*
3.649246	0.891	0.286	2.78E-67	GC B	*MARCKSL1*
2.216658	0.673	0.144	2.01E-58	GC B	*RFTN1*
2.50814	0.752	0.204	1.84E-55	GC B	*LRMP*
2.555376	1	0.748	5.69E-54	GC B	*ACTG1*
2.186426	0.683	0.278	6.36E-25	GC B	*S100A10*
5.865864	0.586	0.061	0	Plasma G	*IGHG4*
4.811712	0.519	0.146	0	Plasma G	*IGHG1*
6.422412	0.358	0.077	1.54E-240	Plasma G	*IGHV5-51*
6.682992	0.25	0.049	3.19E-167	Plasma G	*IGHV1-69D*
5.415041	0.206	0.034	5.76E-143	Plasma G	*IGLV6-57*
5.013435	0.148	0.024	6.03E-98	Plasma G	*IGKV1-27*
4.64899	0.107	0.02	4.64E-63	Plasma G	*IGKV1-16*
5.542505	0.123	0.027	5.88E-63	Plasma G	*IGKV1-12*
4.806145	0.181	0.063	2.61E-56	Plasma G	*IGKV3-15*
5.241723	0.164	0.104	2.66E-12	Plasma G	*IGKV1-39*
5.030855	0.789	0.07	1.14E-246	Plasma A	*IGHA2*
2.973739	0.994	0.287	2.10E-134	Plasma A	*TXNDC5*

3.73988	1	0.388	2.82E-115	Plasma A	*JCHAIN*
4.015661	0.807	0.159	3.58E-114	Plasma A	*IGHA1*
2.678921	1	0.497	3.81E-97	Plasma A	*HSP90B1*
2.965819	1	0.638	5.41E-97	Plasma A	*PPIB*
3.940509	0.106	0.016	8.29E-14	Plasma A	*IGKV2D-30*
3.337215	0.13	0.028	1.03E-09	Plasma A	*IGLV2-8*
4.117266	0.155	0.05	0.000194	Plasma A	*IGKV3OR2-268*
3.364274	0.106	0.039	0.328936	Plasma A	*IGKV2-30*

Table S6. Top 10 marker genes for macrophage subclusters.

avg_log2FC	pct.1	pct.2	p_val_adj	cluster	gene
3.686346	0.719	0.083	5.10E-94	*CXCL9*$^+$ Macrophage	*MMP9*
3.464273	0.702	0.096	6.26E-85	*CXCL9*$^+$ Macrophage	*UBD*
3.297105	0.538	0.055	1.01E-69	*CXCL9*$^+$ Macrophage	*PLA2G2D*
4.513236	0.415	0.025	2.46E-64	*CXCL9*$^+$ Macrophage	*PTGDS*
2.056352	0.865	0.542	1.72E-33	*CXCL9*$^+$ Macrophage	*LYZ*
2.102425	0.444	0.095	2.85E-31	*CXCL9*$^+$ Macrophage	*RARRES1*
2.058084	0.333	0.066	2.34E-22	*CXCL9*$^+$ Macrophage	*CXCL9*
1.934913	0.456	0.16	7.46E-20	*CXCL9*$^+$ Macrophage	*NR1H3*
1.874041	0.24	0.042	5.08E-17	*CXCL9*$^+$ Macrophage	*SPARC*
2.330293	0.111	0.004	1.02E-16	*CXCL9*$^+$ Macrophage	*CHIT1*
3.236649	0.438	0.009	1.91E-87	*CXCL13*$^+$ Macrophage	*HSD11B1-AS1*
1.926957	0.995	0.997	1.54E-66	*CXCL13*$^+$ Macrophage	*FTH1*
2.901873	0.42	0.04	3.18E-54	*CXCL13*$^+$ Macrophage	*CXCL13*
1.795338	0.237	0.01	2.29E-38	*CXCL13*$^+$ Macrophage	*FEZ1*
1.840085	0.265	0.021	3.18E-36	*CXCL13*$^+$ Macrophage	*PPM1N*
2.400176	0.233	0.014	2.09E-33	*CXCL13*$^+$ Macrophage	*CD5L*
2.262952	0.402	0.11	2.57E-27	*CXCL13*$^+$ Macrophage	*GNG2*
2.082582	0.502	0.185	3.44E-27	*CXCL13*$^+$ Macrophage	*EBI3*
2.245313	0.507	0.237	1.39E-21	*CXCL13*$^+$ Macrophage	*BASP1*
1.930194	0.256	0.058	8.85E-17	*CXCL13*$^+$ Macrophage	*S100B*
1.875055	0.596	0.219	1.04E-39	*TREM2*$^+$ Macrophage	*ALOX5AP*
1.305604	0.326	0.048	1.63E-34	*TREM2*$^+$ Macrophage	*OLFML3*
1.442198	0.674	0.339	5.08E-30	*TREM2*$^+$ Macrophage	*TREM2*
1.599127	0.646	0.274	7.61E-27	*TREM2*$^+$ Macrophage	*SPP1*
1.510691	0.316	0.072	6.24E-24	*TREM2*$^+$ Macrophage	*HTRA1*
1.023276	0.786	0.531	1.35E-18	*TREM2*$^+$ Macrophage	*LGALS1*
1.243387	0.635	0.427	2.27E-16	*TREM2*$^+$ Macrophage	*YWHAH*
1.23556	0.274	0.13	0.000112	*TREM2*$^+$ Macrophage	*APOC2*
1.195068	0.154	0.075	0.633321	*TREM2*$^+$ Macrophage	*CCL3L1*
1.444082	0.14	0.083	1	*TREM2*$^+$ Macrophage	*CCL4L2*
2.285953	0.754	0.261	1.96E-63	*FOLR2*$^+$ Macrophage	*SELENOP*
1.807421	0.434	0.123	1.18E-31	*FOLR2*$^+$ Macrophage	*SLC40A1*
1.188681	0.768	0.475	2.35E-31	*FOLR2*$^+$ Macrophage	*MS4A6A*

1.44214	0.627	0.308	3.59E-29	*FOLR2*$^+$ Macrophage	*MS4A4A*
1.062625	0.812	0.596	2.49E-25	*FOLR2*$^+$ Macrophage	*CTSC*
1.043123	0.517	0.261	3.02E-16	*FOLR2*$^+$ Macrophage	*CD163*
1.210713	0.307	0.104	2.65E-15	*FOLR2*$^+$ Macrophage	*MRC1*
1.524167	0.243	0.101	1.37E-07	*FOLR2*$^+$ Macrophage	*PDK4*
1.607673	0.257	0.118	3.67E-06	*FOLR2*$^+$ Macrophage	*RNASE1*
1.218956	0.29	0.188	0.39811	*FOLR2*$^+$ Macrophage	*MT1X*
2.248677	0.51	0.021	7.39E-88	*FCN1*$^+$ Macrophage	*FCN1*
3.544452	0.624	0.082	1.48E-66	*FCN1*$^+$ Macrophage	*S100A8*
2.56108	0.953	0.392	2.45E-62	*FCN1*$^+$ Macrophage	*S100A6*
2.124769	0.926	0.391	7.67E-48	*FCN1*$^+$ Macrophage	*S100A4*
2.186326	0.812	0.268	2.17E-47	*FCN1*$^+$ Macrophage	*EMP3*
2.047779	0.933	0.405	2.29E-45	*FCN1*$^+$ Macrophage	*S100A10*
1.731137	0.805	0.256	1.62E-41	*FCN1*$^+$ Macrophage	*NEAT1*
2.197236	0.537	0.13	2.40E-30	*FCN1*$^+$ Macrophage	*CRIP1*
2.384063	0.745	0.297	2.01E-27	*FCN1*$^+$ Macrophage	*S100A9*
1.65387	0.611	0.209	1.49E-25	*FCN1*$^+$ Macrophage	*FBP1*

Table S7. Top 10 marker genes for CD4$^+$ T cell subclusters.

avg_log2FC	pct.1	pct.2	p_val_adj	cluster	gene
1.406359	0.789	0.371	0	Tn	*CCR7*
0.908899	0.734	0.411	0	Tn	*LEF1*
0.882853	0.253	0.025	0	Tn	*AIF1*
0.835967	0.822	0.464	0	Tn	*SELL*
0.77745	0.921	0.762	0	Tn	*SNHG29*
0.741435	0.307	0.086	0	Tn	*ACTN1*
0.715832	0.999	0.995	0	Tn	*RPS13*
0.691438	0.818	0.568	0	Tn	*TCF7*
0.685324	0.99	0.941	0	Tn	*EEF1B2*
0.728004	0.619	0.397	1.59E-240	Tn	*PRKCQ-AS1*
0.665955	0.453	0.238	1.59E-164	*LIMS1*$^+$ Tm	*LIMS1*
0.526492	0.253	0.112	2.34E-110	*LIMS1*$^+$ Tm	*PASK*
0.454811	0.776	0.607	2.88E-110	*LIMS1*$^+$ Tm	*TCF7*
0.406143	0.816	0.718	7.36E-86	*LIMS1*$^+$ Tm	*FYB1*
0.415447	0.839	0.745	1.15E-85	*LIMS1*$^+$ Tm	*GIMAP4*
0.515396	0.578	0.422	4.90E-82	*LIMS1*$^+$ Tm	*AQP3*
0.458726	0.664	0.521	5.06E-82	*LIMS1*$^+$ Tm	*RNASET2*
0.414941	0.49	0.353	2.74E-59	*LIMS1*$^+$ Tm	*GPR183*
0.378174	0.427	0.292	7.84E-59	*LIMS1*$^+$ Tm	*TRADD*
0.489293	0.518	0.42	3.03E-43	*LIMS1*$^+$ Tm	*ITGA4*
0.933804	0.96	0.794	0	*TIMP1*$^+$ Tm	*CRIP1*
0.877686	0.798	0.475	0	*TIMP1*$^+$ Tm	*ANXA1*
0.860526	0.986	0.797	0	*TIMP1*$^+$ Tm	*S100A4*
0.844224	0.785	0.448	0	*TIMP1*$^+$ Tm	*AHNAK*

0.834688	0.933	0.746	0	*TIMP1*⁺ Tm	*IL7R*
0.810702	0.939	0.71	0	*TIMP1*⁺ Tm	*S100A11*
0.770599	0.92	0.742	0	*TIMP1*⁺ Tm	*S100A10*
0.681022	0.946	0.867	1.45E-284	*TIMP1*⁺ Tm	*VIM*
0.88339	0.521	0.235	7.54E-246	*TIMP1*⁺ Tm	*LGALS1*
0.703638	0.533	0.265	8.67E-223	*TIMP1*⁺ Tm	*TIMP1*
2.180099	0.601	0.057	0	Tem	*GZMK*
1.530265	0.751	0.127	0	Tem	*CCL5*
1.513706	0.614	0.121	0	Tem	*GZMA*
1.116576	0.464	0.118	0	Tem	*LYAR*
0.906344	0.524	0.165	2.60E-288	Tem	*CXCR3*
0.934683	0.937	0.765	2.26E-261	Tem	*IL7R*
0.866335	0.84	0.503	2.63E-223	Tem	*ANXA1*
0.749737	0.318	0.092	1.60E-181	Tem	*HOPX*
0.694212	0.343	0.123	2.43E-146	Tem	*KLRG1*
0.729584	0.382	0.166	1.70E-121	Tem	*PTGER2*
5.244955	0.998	0.048	0	Temra	*NKG7*
4.885738	0.776	0.013	0	Temra	*GNLY*
4.314072	0.972	0.009	0	Temra	*GZMH*
3.727311	0.99	0.138	0	Temra	*CCL5*
3.292965	0.791	0.002	0	Temra	*FGFBP2*
3.258164	0.659	0.003	0	Temra	*GZMB*
2.79818	0.854	0.14	0	Temra	*PRF1*
2.75165	0.937	0.121	0	Temra	*GZMA*
2.665094	0.931	0.183	0	Temra	*CST7*
2.598119	0.681	0.006	0	Temra	*CX3CR1*
1.662254	0.476	0.024	0	*TNFRSF18*⁻ Treg	*FOXP3*
1.436847	0.359	0.028	0	*TNFRSF18*⁻ Treg	*RTKN2*
1.362386	0.408	0.025	0	*TNFRSF18*⁻ Treg	*IKZF2*
1.051805	0.388	0.088	6.87E-294	*TNFRSF18*⁻ Treg	*STAM*
1.029091	0.471	0.135	6.62E-258	*TNFRSF18*⁻ Treg	*TIGIT*
1.296249	0.499	0.178	6.23E-212	*TNFRSF18*⁻ Treg	*HLA-DRB1*
0.98458	0.992	0.929	2.39E-203	*TNFRSF18*⁻ Treg	*IL32*
1.001129	0.637	0.337	2.63E-156	*TNFRSF18*⁻ Treg	*GBP5*
0.94186	0.333	0.112	2.91E-137	*TNFRSF18*⁻ Treg	*LGALS3*
1.007148	0.296	0.095	1.06E-130	*TNFRSF18*⁻ Treg	*HLA-DRB5*
2.212177	0.519	0.049	0	*TNFRSF18*⁺ Treg	*FOXP3*
2.256078	0.284	0.02	2.08E-250	*TNFRSF18*⁺ Treg	*PGA3*
1.964033	0.451	0.057	1.19E-244	*TNFRSF18*⁺ Treg	*RGS1*
2.079042	0.478	0.071	5.49E-227	*TNFRSF18*⁺ Treg	*TNFRSF18*
2.099815	0.572	0.11	1.43E-214	*TNFRSF18*⁺ Treg	*CTLA4*
2.136169	0.622	0.15	4.52E-185	*TNFRSF18*⁺ Treg	*TIGIT*
2.422251	0.581	0.183	5.98E-125	*TNFRSF18*⁺ Treg	*TNFRSF4*
1.882614	0.924	0.738	5.57E-114	*TNFRSF18*⁺ Treg	*CD74*

1.707142	0.824	0.533	1.44E-104	*TNFRSF18*$^+$ Treg	*SRGN*
1.825741	0.503	0.163	4.82E-98	*TNFRSF18*$^+$ Treg	*BATF*
4.008559	0.255	0.003	0	Tfh	*CXCL13*
2.311572	0.905	0.522	0	Tfh	*SRGN*
2.104559	0.534	0.055	0	Tfh	*PDCD1*
1.963433	0.274	0.004	0	Tfh	*IL21*
1.771358	0.296	0.016	0	Tfh	*CD200*
1.650648	0.394	0.023	0	Tfh	*TOX2*
1.705432	0.239	0.022	9.82E-288	Tfh	*NMB*
1.997127	0.84	0.483	2.05E-253	Tfh	*ITM2A*
1.677074	0.295	0.045	2.83E-223	Tfh	*LAG3*
1.703029	0.402	0.117	6.69E-151	Tfh	*DUSP2*
1.537969	0.432	0.018	0	*ISG*$^+$ T	*IFIT1*
1.827629	0.63	0.073	1.29E-245	*ISG*$^+$ T	*OAS1*
2.151571	0.735	0.121	3.51E-214	*ISG*$^+$ T	*MX1*
2.009554	0.673	0.149	5.72E-135	*ISG*$^+$ T	*IFI6*
2.347718	0.79	0.223	8.23E-129	*ISG*$^+$ T	*ISG15*
1.403107	0.623	0.15	1.50E-102	*ISG*$^+$ T	*IRF7*
1.759279	0.895	0.397	1.61E-94	*ISG*$^+$ T	*STAT1*
1.533382	0.595	0.172	9.21E-77	*ISG*$^+$ T	*GBP1*
1.45441	0.907	0.556	1.70E-68	*ISG*$^+$ T	*LY6E*
1.470986	0.72	0.383	1.13E-35	*ISG*$^+$ T	*MT2A*

Table S8. Top 10 marker genes for CD8$^+$ T cell subclusters.

avg_log2FC	pct.1	pct.2	p_val_adj	cluster	gene
2.404635	0.81	0.11	0	Tn	*CCR7*
1.988566	0.772	0.111	0	Tn	*LEF1*
1.756829	0.816	0.178	0	Tn	*SELL*
1.696751	0.672	0.178	0	Tn	*LINC02446*
1.479196	0.824	0.347	0	Tn	*NOSIP*
1.406821	0.508	0.049	0	Tn	*AIF1*
1.339863	0.491	0.042	0	Tn	*MAL*
1.282247	0.456	0.027	0	Tn	*ACTN1*
1.221441	0.791	0.334	0	Tn	*TCF7*
1.12089	0.941	0.677	0	Tn	*LDHB*
2.183947	0.878	0.291	0	Tem	*GZMK*
1.321141	0.924	0.802	1.52E-253	Tem	*CD74*
1.106879	0.712	0.433	5.25E-212	Tem	*COTL1*
0.824711	0.503	0.246	4.34E-131	Tem	*CXCR3*
0.744219	0.162	0.023	4.24E-125	Tem	*IFNG-AS1*
0.832305	0.549	0.343	2.37E-80	Tem	*HLA-DRB1*
0.884621	0.45	0.271	5.72E-78	Tem	*CLDND1*
0.809036	0.41	0.239	5.44E-65	Tem	*DUSP2*
0.699422	0.547	0.383	8.53E-65	Tem	*CD27*

0.782692	0.323	0.173	3.18E-55	Tem	*HLA-DRA*
2.094327	0.627	0.056	0	Trm	*CAPG*
2.557716	0.694	0.2	4.89E-297	Trm	*JUN*
1.805511	0.862	0.46	8.42E-264	Trm	*CKLF*
1.883029	0.87	0.476	3.06E-246	Trm	*CD69*
2.854119	0.541	0.151	1.46E-209	Trm	*FOS*
1.538506	0.545	0.17	7.64E-180	Trm	*SOCS1*
1.544032	0.322	0.052	1.72E-173	Trm	*KLRC1*
1.79437	0.306	0.061	6.33E-138	Trm	*XCL1*
1.918901	0.387	0.181	4.58E-54	Trm	*CCL4L2*
1.724182	0.618	0.422	9.20E-48	Trm	*CCL4*
3.343602	0.559	0.072	0	Temra	*GNLY*
2.830353	0.673	0.046	0	Temra	*FGFBP2*
2.749512	0.785	0.1	0	Temra	*GZMB*
2.508975	0.927	0.212	0	Temra	*GZMH*
2.205374	0.658	0.048	0	Temra	*CX3CR1*
2.018412	0.998	0.626	0	Temra	*NKG7*
1.978567	0.728	0.117	0	Temra	*KLRD1*
1.858978	0.461	0.023	0	Temra	*FCGR3A*
1.745302	0.808	0.289	0	Temra	*EFHD2*
1.598909	0.907	0.442	0	Temra	*PRF1*
2.99933	0.961	0.17	0	MAIT	*KLRB1*
2.766004	0.781	0.05	0	MAIT	*CEBPD*
2.294265	0.719	0.054	0	MAIT	*TRAV1-2*
2.036902	0.705	0.118	0	MAIT	*NCR3*
1.654308	0.463	0.003	0	MAIT	*SLC4A10*
1.404376	0.948	0.587	2.43E-223	MAIT	*IL7R*
1.24121	0.574	0.196	4.87E-174	MAIT	*AQP3*
1.347678	0.149	0.007	2.31E-167	MAIT	*TRBV6-4*
1.21094	0.929	0.611	9.57E-151	MAIT	*LTB*
1.177885	0.204	0.044	2.68E-80	MAIT	*TYROBP*

Table S9. Signature genes used to define functional gene sets.

**signature**	**genes**
**TLS score**	*CCL2, CCL3, CCL4, CCL5, CCL8, CCL18, CCL19, CCL21, CXCL9, CXCL10, CXCL11, CXCL13*
